# Multi-omics analysis of human patient samples identifies key immune factors in *Leptospira* infection

**DOI:** 10.1128/spectrum.00047-26

**Published:** 2026-06-15

**Authors:** Jusail CP, Pallavi Vyas, Huda Noor, Sridhar Kavela, Mansoor A. Kodugade, Nagamani Kammili, Syed M. Faisal

**Affiliations:** 1 Vaccine Immunology Laboratory, National Institute of Animal Biotechnology 378803, Hyderabad, India; 2 Regional Centre for Biotechnology 214253, Faridabad, India; 3 Gandhi Medical College and Hospital, Hyderabad, India; University of North Dakota, Grand Forks, North Dakota, USA

**Keywords:** leptospirosis, multi-omics, transcriptomics, proteomics, host immune response, biomarkers

## Abstract

Leptospirosis is a globally neglected zoonotic disease caused by pathogenic *Leptospira* species and characterized by diverse clinical manifestations. However, the molecular immune responses occurring in circulating human immune cells during acute infection remain incompletely understood. Although several leptospiral virulence determinants have been investigated, a detailed characterization of host immune signaling during human infection is still limited. To characterize host molecular responses during acute leptospirosis, we performed integrated transcriptomic and proteomic profiling of peripheral blood mononuclear cells (PBMCs) from laboratory-confirmed leptospirosis patients and healthy controls. PBMCs were selected because they contain circulating immune cells involved in pathogen recognition and cytokine signaling, enabling focused analysis of host immune responses. Multi-omics network analysis was used to identify conserved immune pathways, and key host factors were further examined using *ex vivo* human whole-blood infection models and *in vitro* infection of human macrophages and monocytes. Transcriptomic profiling identified over 5,800 differentially expressed genes, predominantly upregulated, and associated with inflammatory signaling, including tumor necrosis factor signaling, interferon responses, and cytokine-mediated immune activation. Proteomic analyses detected 362 differentially expressed proteins in clinical PBMC samples and 818 differentially expressed proteins in the experimental infection model, with overlapping proteins defining a conserved host response. Comparative analysis identified 16 consistently upregulated host factors enriched in pathways related to neutrophil activation, hemostasis, and cytokine signaling. Quantitative RT-PCR confirmed increased expression of *SERPINA1, BASP1, ORM1, NAMPT,* and *GCA,* supporting their roles in inflammatory signaling and acute-phase immune responses. Together, these findings reveal conserved immune pathways activated during acute *Leptospira* infection and provide molecular insights into host-pathogen interactions in human leptospirosis.

**IMPORTANCE:**

Leptospirosis is a globally important zoonotic disease caused by bacteria of the genus *Leptospira*. It can lead to severe systemic complications such as kidney failure, lung injury, and multi-organ dysfunction in affected individuals. Accumulating evidence indicates that these severe clinical manifestations are closely associated with dysregulated host immune and inflammatory responses triggered during *Leptospira* infection. However, the molecular mechanisms underlying these immune responses, particularly the transcriptional and proteomic alterations occurring in human immune cells during infection, remain incompletely understood. In this study, we analyzed immune cells from patients with leptospirosis and identified key host molecules and immune pathways activated during infection. Experiments using human blood and immune cell models confirmed the involvement of several inflammatory host factors. These findings improve our understanding of how the human immune system responds to *Leptospira* infection and identify host-response molecules that may help guide future biomarker discovery and therapeutic strategies.

## INTRODUCTION

Leptospirosis is a globally neglected zoonotic disease caused by pathogenic *Leptospira* spp., posing a significant public health threat, particularly in tropical and subtropical regions. Humans typically acquire infections through direct contact with urine from infected animals or indirectly via contaminated water and soil, with outbreaks often associated with flooding or poor sanitation conditions (1, 2). The clinical spectrum of leptospirosis is highly variable, ranging from mild, self-limiting febrile illness to severe complications such as acute kidney injury, liver dysfunction, pulmonary hemorrhage, meningitis, and multi-organ failure, which contribute to high morbidity and mortality in endemic regions (3, 4). Despite extensive studies on leptospiral virulence determinants, the molecular mechanisms underlying human immune responses to *Leptospira* infection, particularly at the level of circulating immune cells, remain poorly understood. In particular, the transcriptional and proteomic changes of circulating immune cells in peripheral blood during acute infection remain poorly characterized, despite their central role in early host defense and systemic inflammatory signaling (1, 5). This knowledge gap limits the development of accurate diagnostics, effective therapeutics, and broadly protective vaccines (6, 7). Antibiotic therapy with doxycycline or penicillin is effective when administered early but often fails to prevent immune-mediated tissue damage in severe cases. Additionally, emerging concerns regarding antimicrobial resistance and the limitations of existing vaccines underscore the urgent need for alternative strategies that target host immune pathways (1, 8).

Host-pathogen interactions in leptospirosis involve complex networks of innate and adaptive immune processes. Pattern-recognition receptors such as Toll-like receptor 2 (TLR2) and TLR4 recognize leptospiral lipoproteins and lipopolysaccharides, triggering inflammatory cascades that culminate in cytokine release and leukocyte activation (9, 10). While such responses are essential for bacterial clearance, uncontrolled inflammation contributes to tissue injury and clinical severity (11). Understanding how the host immune landscape of circulating blood immune cells changes during infection is therefore critical for identifying biomarkers of disease progression and potential therapeutic targets. Traditional investigations have primarily focused on bacterial virulence factors, serovar diversity, and epidemiology (4, 8). Far fewer studies have explored the human host response in depth. Targeting host-directed pathways within circulating leukocyte populations offers a promising approach for improving disease management. Peripheral blood mononuclear cells (PBMCs), comprising monocytes, lymphocytes, and dendritic cells, serve as the primary line of defense against blood-borne pathogens and play a crucial role in early immune recognition, cytokine signaling, and pathogen clearance. Because PBMCs circulate through the bloodstream during the early stages of infection, they provide a valuable window into systemic immune signaling events triggered by *Leptospira* exposure. Dysregulated immune responses in PBMCs have been previously linked to disease severity, with inter-individual variability shaped by genetic, immunological, and pathogen-specific factors (12). In other diseases, such as tuberculosis, sepsis, and dengue, transcriptomic and proteomic profiling of PBMCs has revealed critical insights into immune evasion, transcriptional alterations associated with metabolic pathways, and potential prognostic biomarkers (13–15). However, the mechanisms by which pathogenic *Leptospira* modulate immune pathways within circulating blood immune cells and initiate systemic inflammatory responses in leptospirosis remain poorly understood. Understanding these early molecular and cellular alterations is crucial for identifying host factors that contribute to protective immunity or immunopathology, and for guiding the development of host-directed therapeutic interventions.

With the advent of high-throughput omics technologies, integrated approaches combining transcriptomics, proteomics, and metabolomics have become powerful tools for characterizing host responses to infection (16, 17). Multi-omics analyzes capture system-wide molecular perturbations, allowing the identification of coordinated changes across regulatory layers. When applied to specific immune cell populations such as PBMCs, these approaches enable the identification of coordinated transcriptional and proteomic changes that reflect early host-pathogen interactions. These approaches have been successfully applied to other infectious diseases, such as tuberculosis, dengue, and COVID-19, to discover biomarkers, predict clinical outcomes, and reveal mechanistic insights into immune dysfunction (18–20). However, comparable multi-omics studies in human leptospirosis are scarce, with most data derived from animal models or *in vitro* systems (21).

To address this, in the present study, we employed an integrative high-throughput multi-omics approach, combining RNA sequencing (RNA-seq) and liquid chromatography-tandem mass spectrometry (LC-MS/MS) analyses of PBMCs from clinically confirmed leptospirosis patient samples to characterize the molecular responses of circulating immune cells during acute infection. In parallel, an *ex vivo* whole-blood infection model using PBMCs infected with different pathogenic serovars of *Leptospira* was employed to mimic the early bloodstream phase of infection. This combined strategy allowed us to map transcriptomic and proteomic changes, identify conserved host immune pathways, and uncover candidate host factors modulated during *Leptospira* infection. By integrating clinical and experimental data, we dissected host-pathogen interactions to identify potential biomarkers and inform the development of novel host-directed therapeutic and preventive strategies against leptospirosis.

## MATERIALS AND METHODS

### Ethics statement and sample collection

Human blood samples were collected from leptospirosis-positive patients (*n* = 2) and healthy volunteers (*n* = 2) at Gandhi Medical College, Hyderabad, India, following approval from the Institutional Ethics Committee (IEC/GMC/2021/03/25). Clinical diagnosis was confirmed by IgM ELISA and qRT-PCR targeting the *LipL32* gene (see Table S1 at https://doi.org/10.5281/zenodo.17519428). Age- and sex-matched volunteers without a history of recent febrile illness served as healthy controls. PBMCs) were isolated from all samples using Histopaque-1077 (Sigma-Aldrich) density gradient centrifugation. The buffy coat was collected, washed three times with phosphate-buffered saline (PBS), and aliquoted for RNA isolation (RNA-seq and qRT-PCR) and protein extraction (LC-MS/MS).

### *Leptospira* strains and culture conditions

Pathogenic *Leptospira interrogans* serovars Icterohaemorrhagiae strain RGA, Pomona, and Hardjo were maintained in Ellinghausen-McCullough-Johnson-Harris (EMJH) basal medium supplemented with 10% EMJH enrichment (BD Difco). Cultures were maintained aerobically at 28°C without agitation and harvested at mid-log phase (10⁸–10⁹ cells/mL). For infection assays, bacteria were pelleted at 9,000 × g for 5 min, washed three times with sterile PBS (pH 7.4), and resuspended in PBS or antibiotic-free RPMI-1640. Cell density was quantified using a Petroff-Hausser chamber under dark-field microscopy.

### Cell culture

THP-1 monocytes (ATCC) were maintained in RPMI-1640 medium (Sigma) supplemented with 10% fetal bovine serum (FBS; Gibco) and 1 × antibiotic-antimycotic solution (Gibco). For macrophage differentiation, THP-1 cells were treated with 10 ng/mL phorbol 12-myristate 13-acetate (PMA; Sigma-Aldrich) for 48 h, followed by a 24 h rest in antibiotic- and PMA-free medium. All cultures were maintained at 37°C with 5% CO_2_ supplementation.

### *In vitro* infection models

To comprehensively characterize the host response to *Leptospira*, we employed three experimental systems: human whole blood, THP-1-derived macrophages, and THP-1 monocytes. For proteomic analysis, human whole blood from healthy volunteers was diluted 1:1 with antibiotic-free RPMI-1640 medium and infected with three pathogenic serovars of *L. interrogans* (Pomona, Icterohaemorrhagiae, and Hardjo; 1 × 10⁸ bacteria/mL) for 24 h at 37°C with 5% CO_2_. Uninfected diluted blood served as a control. PBMCs were then isolated for LC-MS/MS analysis. For transcriptional analysis, all three models were stimulated with live *L. interrogans* (serovar Icterohaemorrhagiae strain RGA; 1 × 10⁸ bacteria/mL), heat-killed *Leptospira* (HK-Lep; 1 × 10⁸ bacteria/mL), or *E. coli* lipolysaccharide (100 ng/mL) for 24 h. To further investigate response dynamics, macrophages were infected at varying multiplicities of infection (MOI 1, 10, 100, and 1,000) and over different time points (4, 12, 24, and 48 h). To evaluate paracrine signaling effects, the supernatant from macrophages infected with live and heat-killed *L. interrogans* serovar Icterohaemorrhagiae strain RGA (24 h, MOI 100) was centrifuged twice (9,000 rpm, 5 min) to remove bacteria and transferred to fresh macrophage cultures for 24 or 48 h. Recipient cells were collected for qRT-PCR analysis to determine indirect gene regulation mediated by soluble factors.

### RNA extraction, sequencing, and bioinformatic analysis

Total RNA was extracted from PBMCs using TRIzol reagent (Invitrogen) according to the manufacturer’s instructions. RNA concentration and purity were measured using a NanoDrop 1000 and a Qubit 4.0 fluorometer (Thermo Fisher Scientific) with the RNA HS Assay Kit (Thermo Fisher Scientific). RNA integrity was then assessed using a TapeStation 4150 system (Agilent Technologies) with High Sensitivity RNA ScreenTape (Agilent Technologies) to obtain RNA integrity number (RIN) values. Libraries were constructed using the NEBNext Ultra II Directional RNA Library Prep Kit (New England Biolabs) according to the manufacturer’s instructions and quantified using the Qubit 4.0 fluorometer with the DNA HS Assay Kit (Thermo Fisher Scientific), and insert size distribution was analyzed on the TapeStation 4150 system using High Sensitivity D1000 ScreenTape (Agilent Technologies). Each PBMC sample was sequenced in duplicate to generate technical replicates for transcriptomic analysis. Sequencing was performed on the Illumina NovaSeq 6000 platform (150 bp paired-end). Raw sequencing data were deposited in the NCBI SRA repository under accession number PRJNA1358538.

Raw FASTQ reads were quality-checked using FastQC v0.11.9 with default parameters. Raw reads were preprocessed using Fastp v0.20.1 with the following parameters: --length_required 50, --correction, --trim_poly_g, and—qualified_quality_phred 30 (22). Processed reads were reevaluated for quality using FastQC, and the results were summarized using MultiQC (23). Processed reads were aligned to the STAR-indexed human reference genome (GRCh38) using STAR v2.7.9a with parameters: --outSAMtype BAM SortedByCoordinate, --outSAMattributes NH HI AS NM MD, --outFilterScoreMinOverLread 0.5, and—outFilterMatchNminOverLread 0.5 (24). Ribosomal RNA (rRNA) and transfer RNA (tRNA) features were removed from the GTF annotation file prior to alignment. Gene counts were generated using featureCounts v0.46.1, and differential gene expression analysis was performed with DESeq2 to identify significantly differentially expressed genes (DEGs) using Benjamini–Hochberg adjusted *P* values (FDR < 0.05 and |log2FoldChange| ≥ 1.5) (25).

The complete set of protein-coding RNA was subjected to GSEA using the hallmark gene set database. Additionally, overrepresentation analysis was performed using the EnrichR web tool on DEGs to identify significantly enriched Kyoto Encyclopedia of Genes and Genomes (KEGG) pathways and Gene Ontology (GO) terms, including biological process (BP), molecular function (MF), and cellular component (CC) categories.

### Protein extraction, LC-MS/MS, and proteomic analysis

Protein extraction and sample preparation for LC-MS/MS were performed using previously reported methods with minor modifications (26). The same PBMC samples used for transcriptomic analysis were subjected to proteomic profiling using LC-MS/MS technical replicates (triplicate for control samples and quadruplicate for infected samples). Briefly, proteins were extracted from PBMCs using lysis buffer (50 mM ammonium bicarbonate, 1% sodium deoxycholate) with heating (100°C, 5 min) and sonication. After centrifugation (16,000 × g, 30 min), the supernatants were collected and quantified using the BCA assay (Thermo Fisher). Proteins (100 µg) were reduced (10 mM DTT, 56°C, 1 h), alkylated (20 mM iodoacetamide, RT, 20 min in the dark), and digested with trypsin/Lys-C (Promega; 1:30 enzyme-to-protein ratio, 37°C, overnight). Peptides were purified using C18 columns (Pierce), vacuum-dried, and resuspended in 0.1% formic acid. LC-MS/MS analysis was performed on an Ultimate 3000 RSLC nano system coupled to a Q Exactive HF mass spectrometer (Thermo Scientific) over a 90 min gradient. The mass spectrometry proteomics data have been deposited in the ProteomeXchange Consortium via the PRIDE partner repository with the data set identifier PXD070502 (27). The proteomic data were processed using Proteome Discoverer software (v2.4.1.15, Thermo Fisher Scientific) in label-free quantification mode, following a standard workflow. Spectra were analyzed using the SequestHT search algorithm with default settings. A database search was performed against the trypsin-digested human proteome (SwissProt, reviewed) downloaded from UniProt, with a FASTA file of common contaminants included for quality control. Normalized protein abundance values were exported after filtering out contaminants and imported into the Perseus platform for statistical analysis (28). Proteins with at least 70% valid values in one or more sample groups were retained for further analysis. Differentially expressed proteins (DEPs) were identified using a two-tailed Student’s *t*-test with a significance threshold of *P* < 0.05 and |log2FoldChange| ≥ 1.

### Integrated bioinformatic analysis

To gain a comprehensive understanding of the host response, we performed integrated bioinformatic analysis of both transcriptomic and proteomic data. Specifically, common DEGs and DEPs were identified and subjected to Spearman correlation analysis using GraphPad Prism to assess concordance between RNA and protein expression levels. Additionally, Reactome pathway analysis and disease enrichment analysis were performed using the EnrichR tool, which utilized the DisGeNET data set, to investigate potential disease associations. Finally, protein-protein interaction (PPI) networks were constructed for the commonly upregulated factors using the STRING database with a high-confidence interaction score threshold of 0.7. The resulting networks were visualized and analyzed using Cytoscape software to identify key hub proteins and functional modules.

### Quantitative RT-PCR validation

To validate the selected host factors identified through multi-omics analysis, qRT-PCR was performed. Following infection experiments, PBMCs or cells were harvested, and total RNA was extracted using the NucleoSpin RNA Isolation Kit (Takara, Cat# 740955.25) according to the manufacturer’s instructions. RNA purity and concentration were determined using a NanoDrop 2000 spectrophotometer (Thermo Scientific). Complementary DNA (cDNA) was synthesized from 1 µg of total RNA using the PrimeScript 1st Strand cDNA Synthesis Kit (Takara, Cat# 6110A). The qRT-PCR reactions were performed in 10 µL volumes containing 50 ng of cDNA, 0.2 µM of each gene-specific primer (see Table S3 at https://doi.org/10.5281/zenodo.17519428), and iTaq Universal SYBR Green Supermix (BioRad, Cat# 1725124). All reactions were run in duplicate on a CFX Opus 96 real-time PCR system (BioRad). Gene expression was normalized to GAPDH and calculated via the 2^(–ΔΔCt) method. Results are presented as log2 fold changes.

### Cytokine quantification

Supernatant concentrations of IL-6 were measured using human DuoSet ELISA kits (R&D Systems) following the manufacturer’s protocol. Absorbance was measured at 450 nm, and concentrations were calculated from standard curves.

### Statistical analysis

All statistical analyses were performed using GraphPad Prism v10. Data are presented as mean ± SEM from biological replicates (*n* = 2–3, unless specified). For omics data, significance was defined as FDR < 0.05 and log2 fold change ≥1.5 (RNA) or *P* < 0.05 and |FC| ≥ 1 (protein). For qRT-PCR, an unpaired Student’s *t*-test was used with significance at *P < 0.05 and **P < 0.01.

## RESULTS

### Transcriptomic profiling of PBMCs from leptospirosis patients

**Fig 1:**
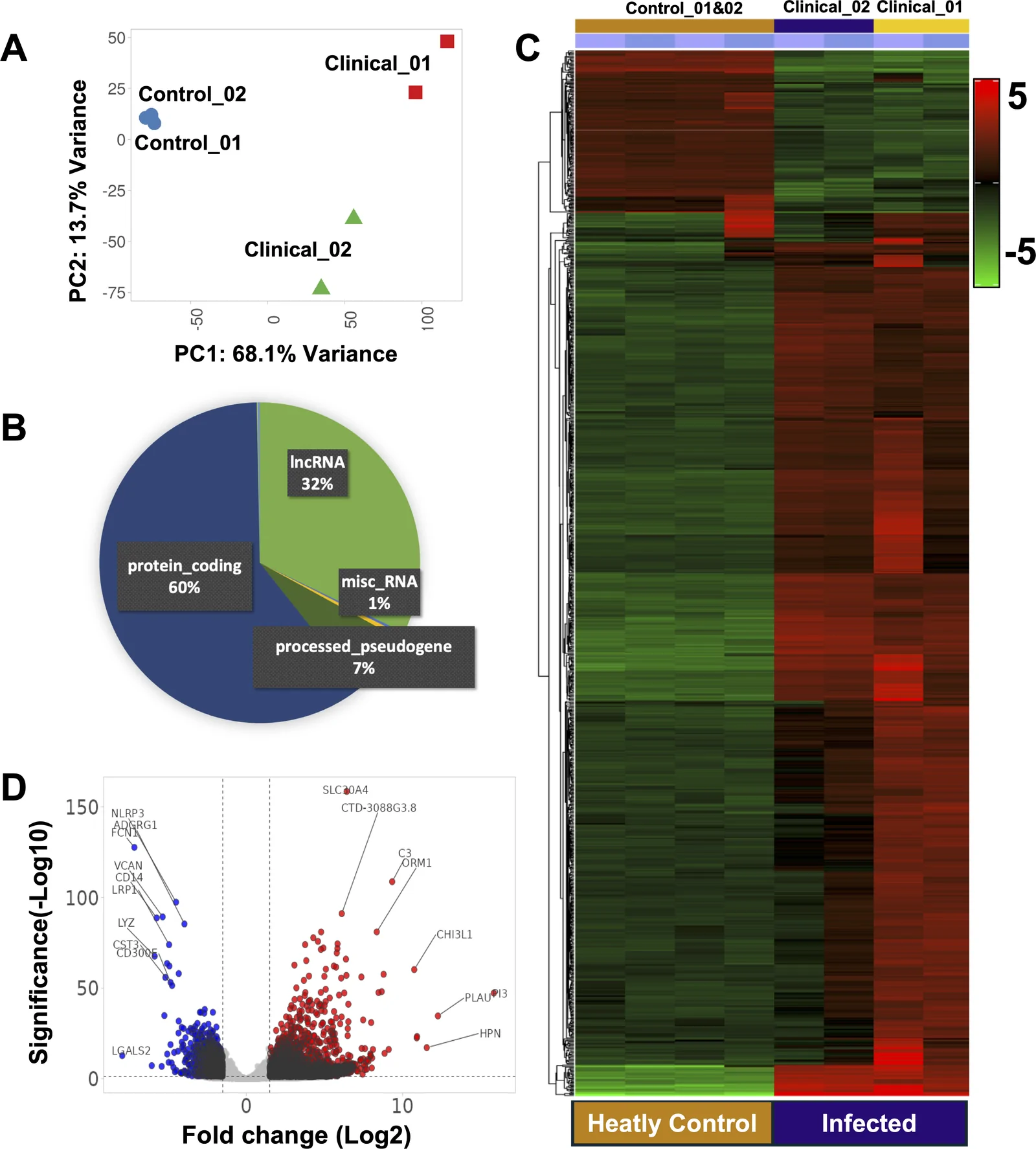
Transcriptomic profiling of PBMCs from acute leptospirosis patients. (**A**) Principal component analysis (PCA) showing global transcriptomic variation and clear separation between healthy controls and leptospirosis clinical samples. Percent variance explained by PC1 and PC2 is indicated. (**B**) RNA biotype distribution of mapped transcriptomic reads, representing proportions of protein-coding, lncRNA, pseudogene, and other RNA categories. (**C**) Hierarchical clustering heatmap of the top 1,000 most variable genes across infected and control samples. Relative expression is shown on a green-to-red scale (lower to higher expression). Black cells indicate missing or low-confidence values. (**D**) Volcano plot of differentially expressed genes (DEGs), displaying log₂ fold change against −log₁₀ adjusted *P* value.

RNA sequencing of PBMCs from leptospirosis patients and healthy controls yielded an average of 35 million high-quality paired-end reads per sample. For ease of comparison, the infected groups were designated as Clinical_01 and Clinical_02. The Principal component analysis (PCA) revealed a distinct separation between the patient and control samples, indicating global transcriptional remodeling during infection (PC1 − 68.1% variance) (Fig. 1A). Approximately 60% of the sequenced RNA was annotated as protein-coding RNA, 32% as long non-coding RNA (lncRNA), 7% as processed pseudogenes, and 1% as miscellaneous RNA (Fig. 1B).

**Fig 2:**
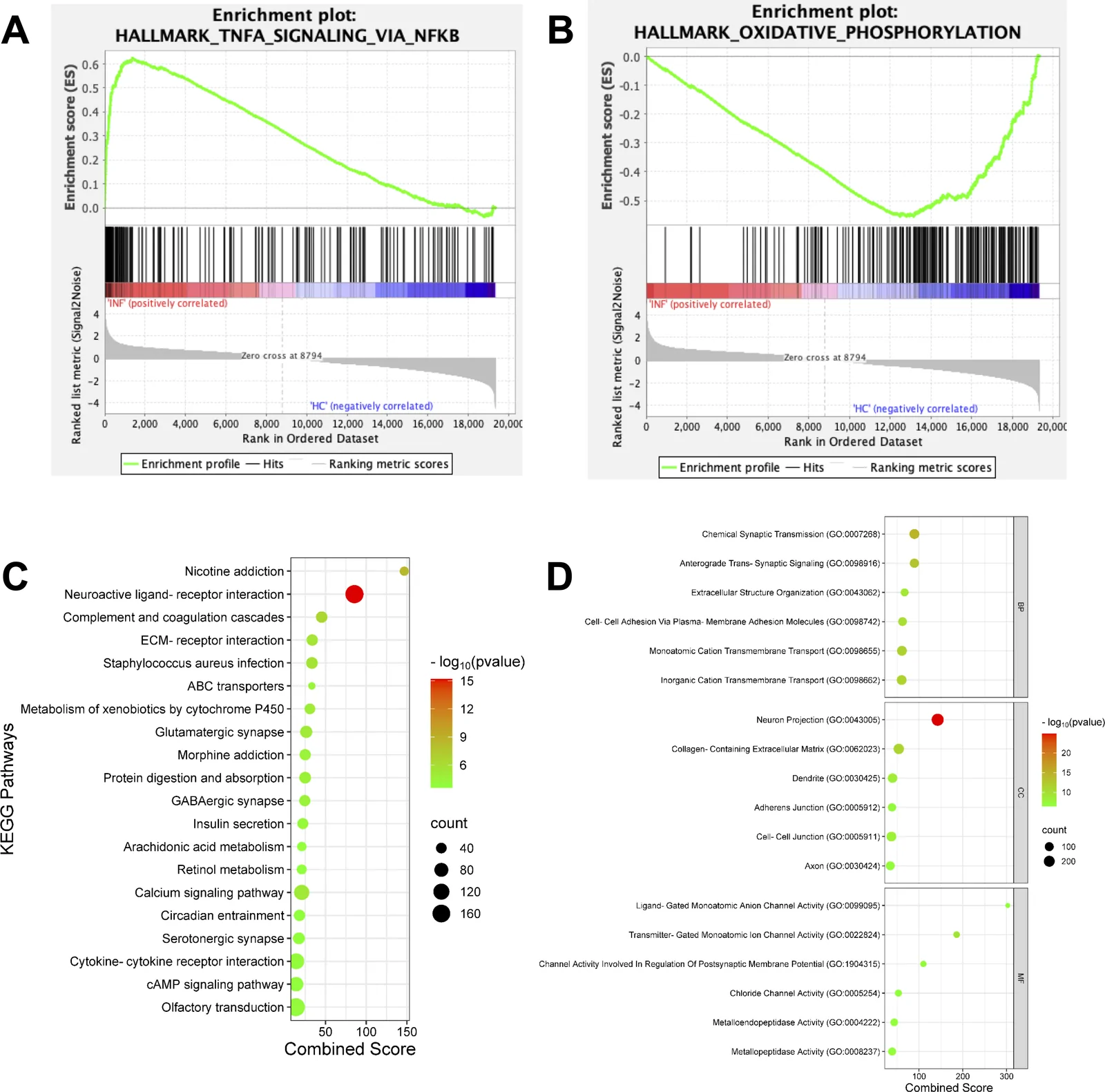
Gene set enrichment and pathway overrepresentation analysis of differentially expressed genes (**A**) GSEA enrichment plot showing significant enrichment of the hallmark_tnfa_signaling_via_nfkb gene set in infected samples. (**B**) GSEA enrichment plot showing enrichment of the hallmark_oxidative_phosphorylation gene set in healthy controls. (**C**) Top 20 KEGG pathways enriched among DEGs identified from clinical samples. (**D**) Top 10 Gene Ontology (GO) categories enriched for DEGs, categorized into biological process (BP), cellular component (CC), and molecular function (MF).

Here, we focused on significantly expressed mRNAs (*P* < 0.05) to identify the DEGs. In hierarchical clustering, it is evident that the uninfected groups clustered together, while the infected individuals exhibited similar clustering, indicating less variability in gene expression between the two infected individuals at the RNA level (Fig. 1C). Compared to the healthy control group, 5,801 genes showed significant changes in expression (log2 fold change >1.5 or <−1.5), with 4,600 genes upregulated and 1,201 genes downregulated in the infected group (Fig. 1D). GSEA revealed a stark contrast in the transcriptional programs of *Leptospira*-infected (INF) and healthy control (HC) groups (see Fig. S1 at https://doi.org/10.5281/zenodo.17519428). We identified 11 hallmark pathways that were significantly enriched in the INF group (FDR < 0.05), predominantly related to inflammatory and antiviral defense, with the top hits being TNFα signaling via NFκB and the interferon α and γ response. Conversely, the HC group showed significant enrichment in nine pathways (FDR < 0.05) fundamental to cellular homeostasis, most notably oxidative phosphorylation, MYC targets V1, and DNA repair, indicating a wholesale shift from routine metabolism to intense immune activation upon infection (Fig. 2A and B and see Table S2 at https://doi.org/10.5281/zenodo.17519428). To further explore the functional implications of these findings, overrepresentation analysis was performed on the DEGs using KEGG pathway and Gene Ontology (GO) analyses. Significantly enriched KEGG pathways included complement and coagulation cascades, ECM-receptor interaction, *Staphylococcus aureus* infection, and cytokine-cytokine receptor interaction. GO analysis revealed significant enrichment in biological processes, such as extracellular structure organization and cell-cell adhesion via plasma membrane adhesion molecules (Fig. 2C and D).

### Differential protein expression in PBMCs during human *Leptospira* infection

**Fig 3:**
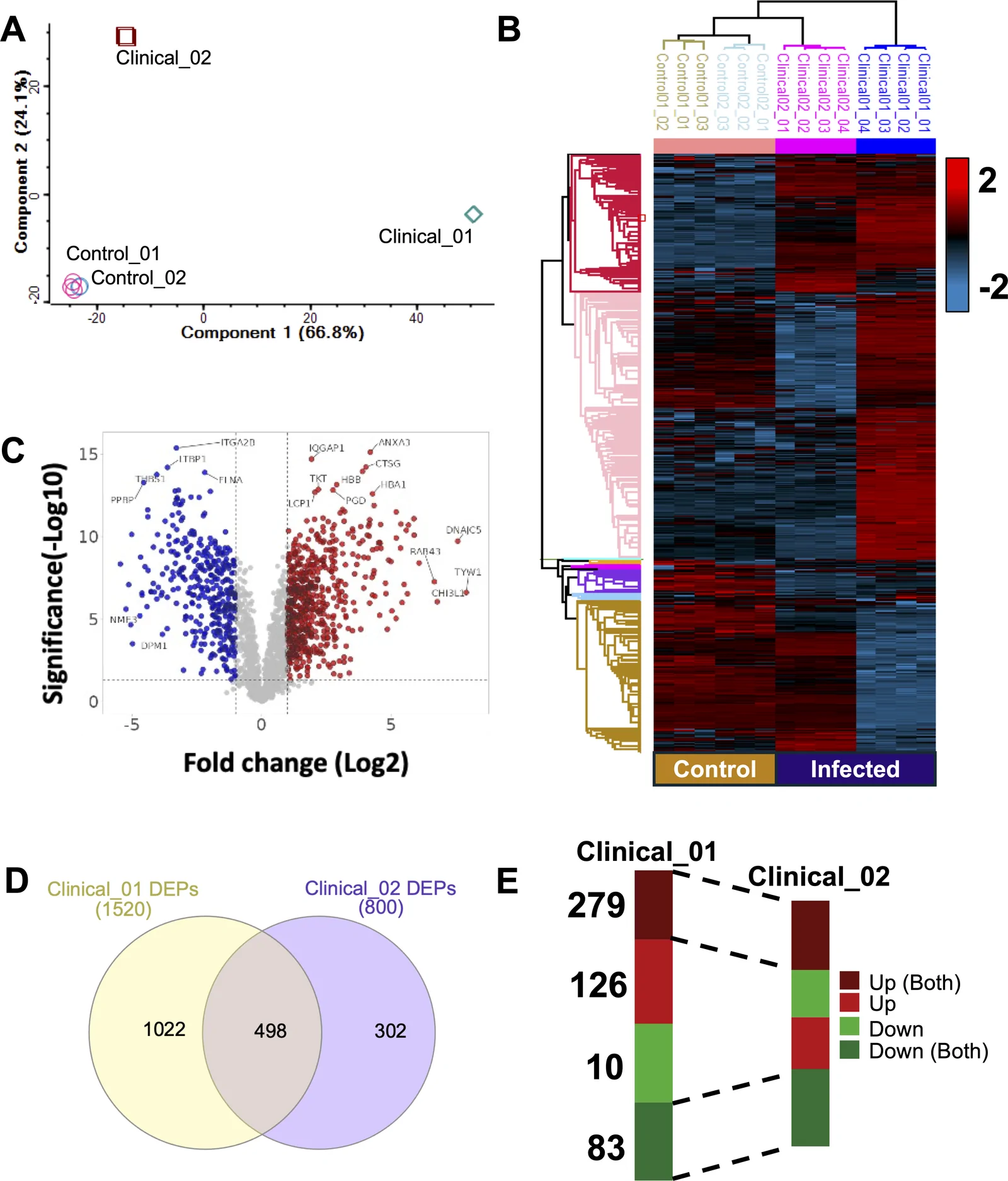
Proteomic alterations in clinical leptospirosis samples. (**A**) PCA plot based on LC-MS/MS proteomic profiles showing clustering of control versus infected samples. (**B**) Heatmap of differentially expressed proteins (DEPs) in infected versus control samples. (**C**) Volcano plot showing distribution of DEPs in Clinical_01 by log₂ fold change and -log₁₀ *P* value. (**D**) Venn diagram showing the overlap of DEPs detected in the two individual infected clinical samples. (**E**) Venn diagram showing shared and unique upregulated/downregulated proteins across the two infected samples.

Following the transcriptional analysis, we performed LC-MS/MS analysis on the same PBMCs to investigate the proteomic changes induced by *Leptospira* infection. The PCA of the proteomic data revealed a distinct separation between infected and uninfected groups. However, unlike the transcriptomic data, the proteomic profiles of the two infected individuals (Clinical_01 and Clinical_02) exhibited greater variability (PC1 − 66.8% variance) compared to the uninfected group (Fig. 3A). To further explore this variability, we generated a clustered heatmap of protein expression profiles. This analysis confirmed that, in contrast to the transcriptomic data, the proteomic expression profiles of the infected individuals showed distinct heterogeneity (Fig. 3B). To account for this variability, we conducted separate analyses to identify DEPs in the Clinical_01 and Clinical_02 samples compared to the uninfected group. In the Clinical_01 sample, 1,520 DEPs were identified, while the Clinical_02 sample showed 800 DEPs, both with significant changes (*P* < 0.05 and log2 fold change ≥ 1) (Fig. 3C). Among these, 498 DEPs were common to both infected individuals, with 279 proteins upregulated and 83 proteins downregulated in both infected samples (Fig. 3D and E).

### Integrated transcriptomic and proteomic profiling of PBMCs from human leptospirosis patients

**Fig 4:**
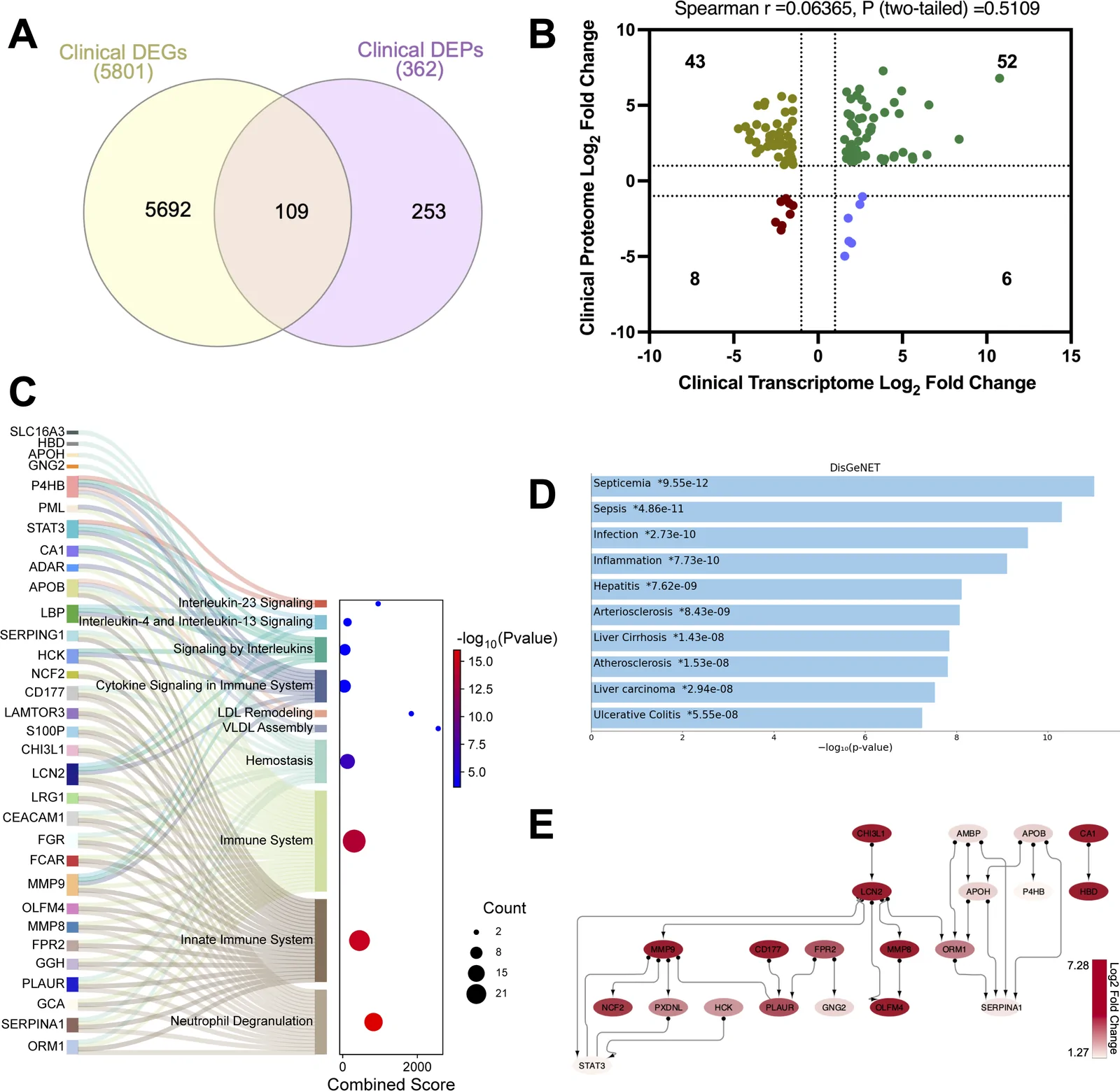
Integrated multi-omics analysis of transcriptomic and proteomic data sets. (**A**) Venn diagram showing overlap between DEGs and DEPs identified from clinical samples. (**B**) Correlation scatter plot comparing log₂ fold change values of overlapping DEGs and DEPs. (**C**) Reactome pathway enrichment of the 52 commonly upregulated molecules, highlighting top pathways. (**D**) DisGeNET disease enrichment of the 52 upregulated factors. (**E**) Protein-protein interaction (PPI) network of upregulated molecules constructed using STRING, displaying interaction clusters.

Integrating transcriptomic and proteomic data provides a comprehensive understanding of the host response to *Leptospira* infection, enhancing confidence in the identification of differentially expressed factors at both the RNA and protein levels. Since the samples used for both transcriptomics and proteomics analyses were derived from the same individuals, we merged the data sets and identified 109 host factors that were differentially expressed at both the RNA and protein levels. Among these, 52 factors were consistently upregulated, and eight were consistently downregulated in both data sets (Fig. 4A and B). Reactome pathway analysis of the 52 upregulated factors revealed significant enrichment in pathways related to neutrophil degranulation, the innate immune system, hemostasis, and cytokine signaling in the immune system (Fig. 4C). To explore the disease associations of these factors, we utilized the DisGeNET data set, which identified conditions such as sepsis, hepatitis, arteriosclerosis, and liver cirrhosis as significantly enriched (Fig. 4D). To further investigate the functional interactions among these factors, we constructed a PPI network using the STRING database with a high-confidence interaction score threshold of 0.7. Visualization of the network in Cytoscape revealed that 21 of the 52 proteins exhibited strong interactions with each other, highlighting potential key players in the host response to *Leptospira* infection (Fig. 4E).

### Differentially expressed proteins in human PBMCs infected with three different serovars of *Leptospira*

**Fig 5:**
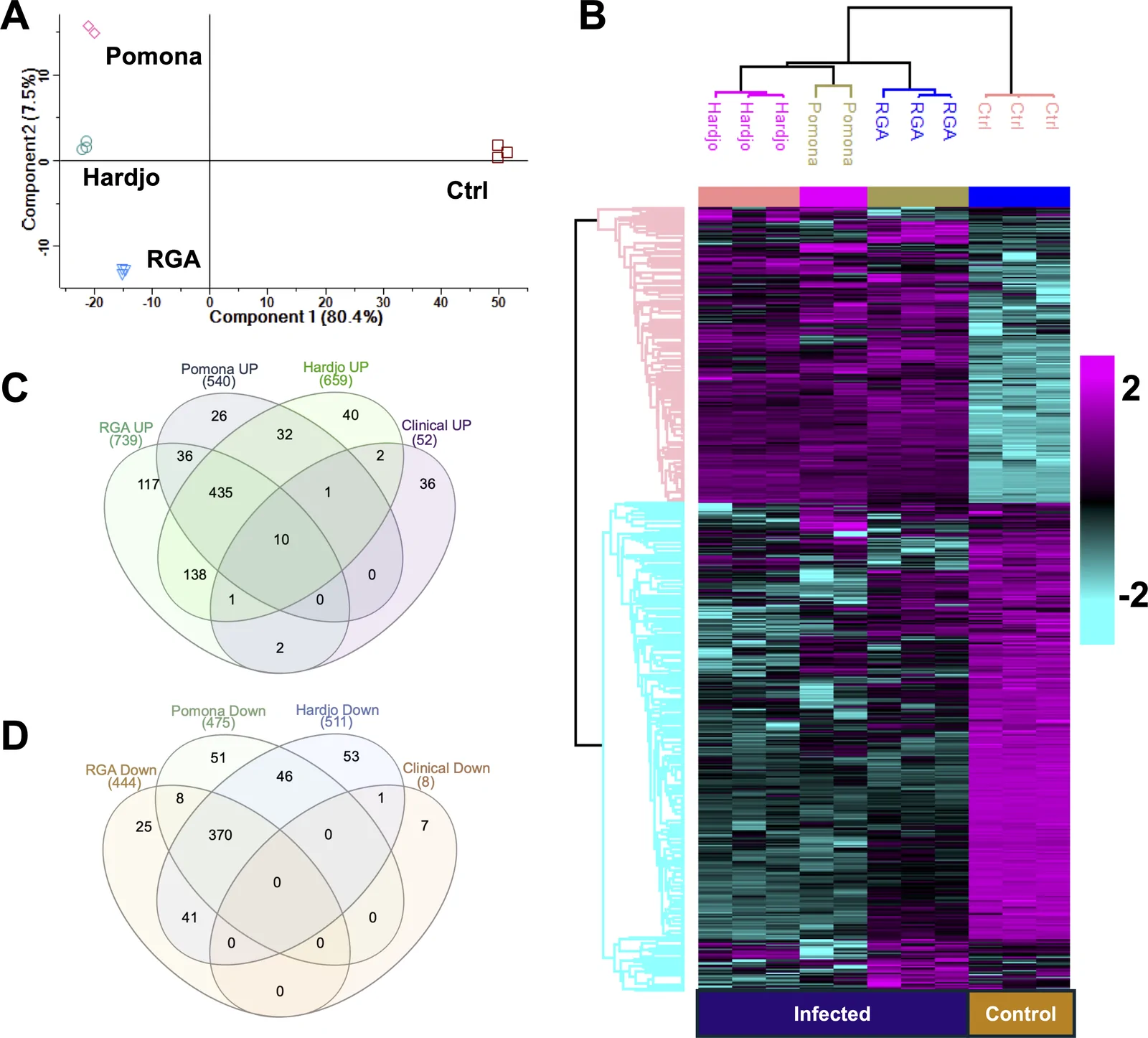
Proteomic response of human whole blood infected with three *Leptospira* serovars. (**A**) PCA plot showing separation between control and samples infected with *L. interrogans* serovars Pomona, Hardjo, and RGA. (**B**) Hierarchical clustering heatmap of DEPs across control and serovar-infected whole blood samples. (**C and D**) Venn diagrams comparing DEPs shared between clinical samples and whole-blood infection models, categorized by upregulated and downregulated proteins.

Due to the limited availability of fresh clinical samples, we employed an *in vitro* whole-blood infection model to enhance confidence in the factors identified from clinical samples. This model closely mimics the physiological conditions of *Leptospira* infection, as the bacteria typically persist in the bloodstream for about a week before disseminating to target organs. To comprehensively investigate the host response, we infected healthy human whole blood with three different serovars of *L. interrogans* (Icterohaemorrhagiae strain RGA, Pomona, and Hardjo) for 24 h. Uninfected whole blood samples served as controls. Following infection, PBMCs were isolated and processed for LC-MS/MS analysis. The PCA revealed a distinct separation between infected and uninfected groups, with the infected group showing significant differences (PC1 − 80.4% variance) (Fig. 5A). To further explore variability, we generated a clustered heatmap of protein expression profiles for three different infected serovars, along with the uninfected group. This analysis confirmed that, in contrast to the uninfected group, the infected group, regardless of serovar, exhibited a similar pattern of expression, clustering together (Fig. 5B). In total, 818 proteins were identified as significantly differentially expressed (*P* < 0.05 and log2 fold change ≥1), regardless of the serovar involved in the infection (see Fig. S2A at https://doi.org/10.5281/zenodo.17519428). Those proteins were known to be involved in pathways related to innate immune systems, protein metabolism, and vesicle-mediated transport (see Fig. S2B at https://doi.org/10.5281/zenodo.17519428). This analysis underscores the shared host response to *Leptospira* infection across different serovars, providing valuable insights into the common molecular mechanisms underlying the disease.

### Distinct factors identified from clinical samples and the whole blood model

The primary goal of this study was to identify critical host factors modulated during *Leptospira* infection. To achieve this, we compared the upregulated and downregulated proteins from the whole-blood proteomics data with the factors identified through conjoint analysis of clinical data. Among the 52 upregulated factors identified from the clinical samples, 10 proteins were commonly upregulated in the whole blood model, while six proteins were uniquely associated with specific serovar infections. These factors were selected for further validation and analysis as potential key players in the host response to *Leptospira* infection (Fig. 5C). In contrast, analysis of downregulated factors revealed no common proteins between the whole-blood model and clinical samples across all serovars. However, one protein, TESC, was identified to be downregulated in samples infected with the Hardjo serovar (Fig. 5D).

### Validation and characterization of core host response factors

**Fig 6:**
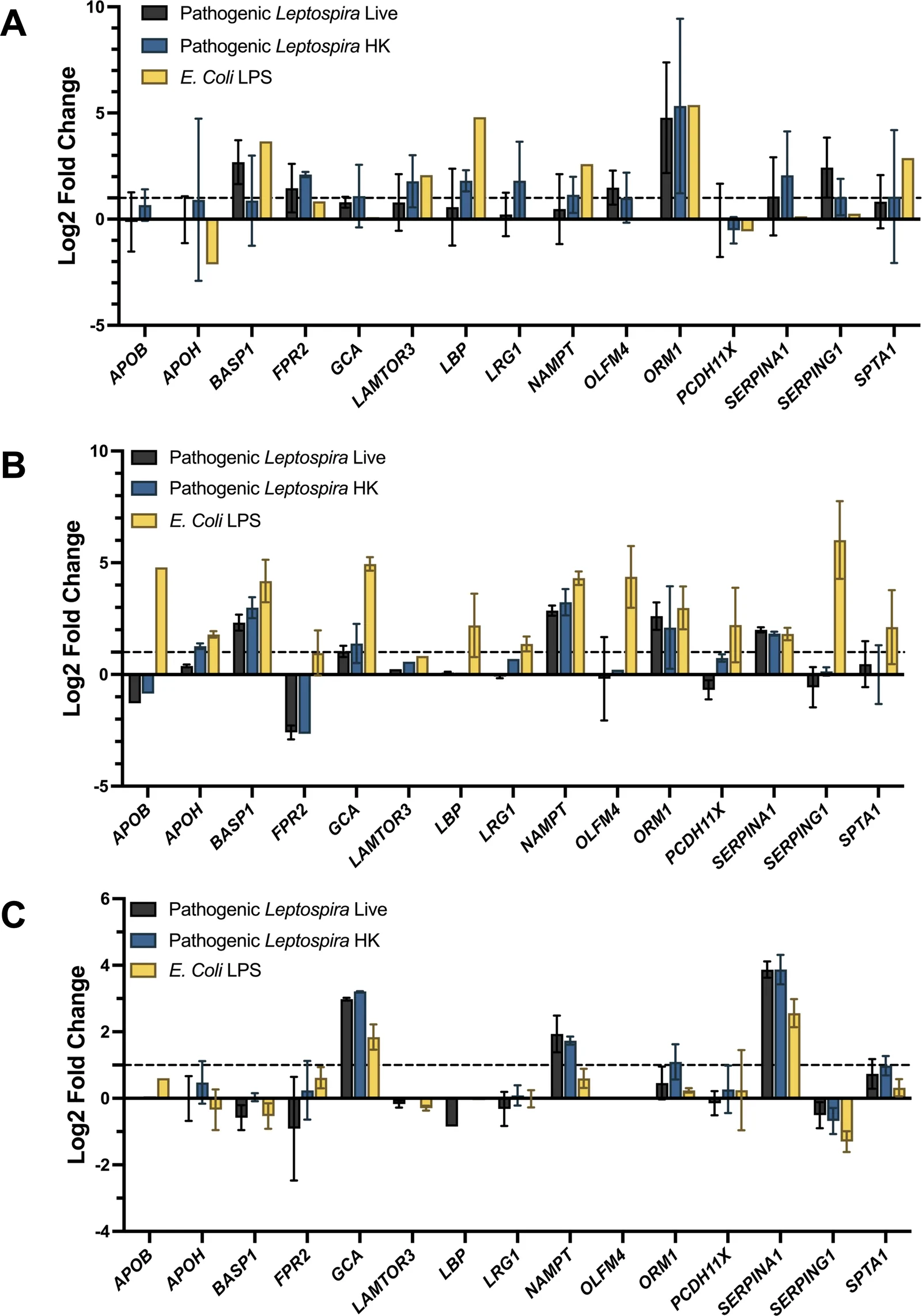
Host immune gene expression in human whole blood, THP-1 macrophages, and THP-1 monocytes. qRT-PCR quantification of 15 candidate host response genes (*APOB, APOH, BASP1, FPR2, GCA, LAMTOR3, LBP, LRG1, NAMPT, OLFM4, ORM1, PCDH11X, SERPINA1, SERPING1,* and *SPTA1*) following 24 h exposure to live pathogenic *Leptospira*, heat-killed *Leptospira* (HK-Lep), or *E. coli* LPS from (**A**) Human whole blood. (**B**) THP-1 macrophages. (**C**) THP-1 monocytes. Bars represent log₂ fold change (ΔΔCt) ±SEM from 2-3 biological replicates. The dotted line indicates the 2-fold threshold.

**Fig 7:**
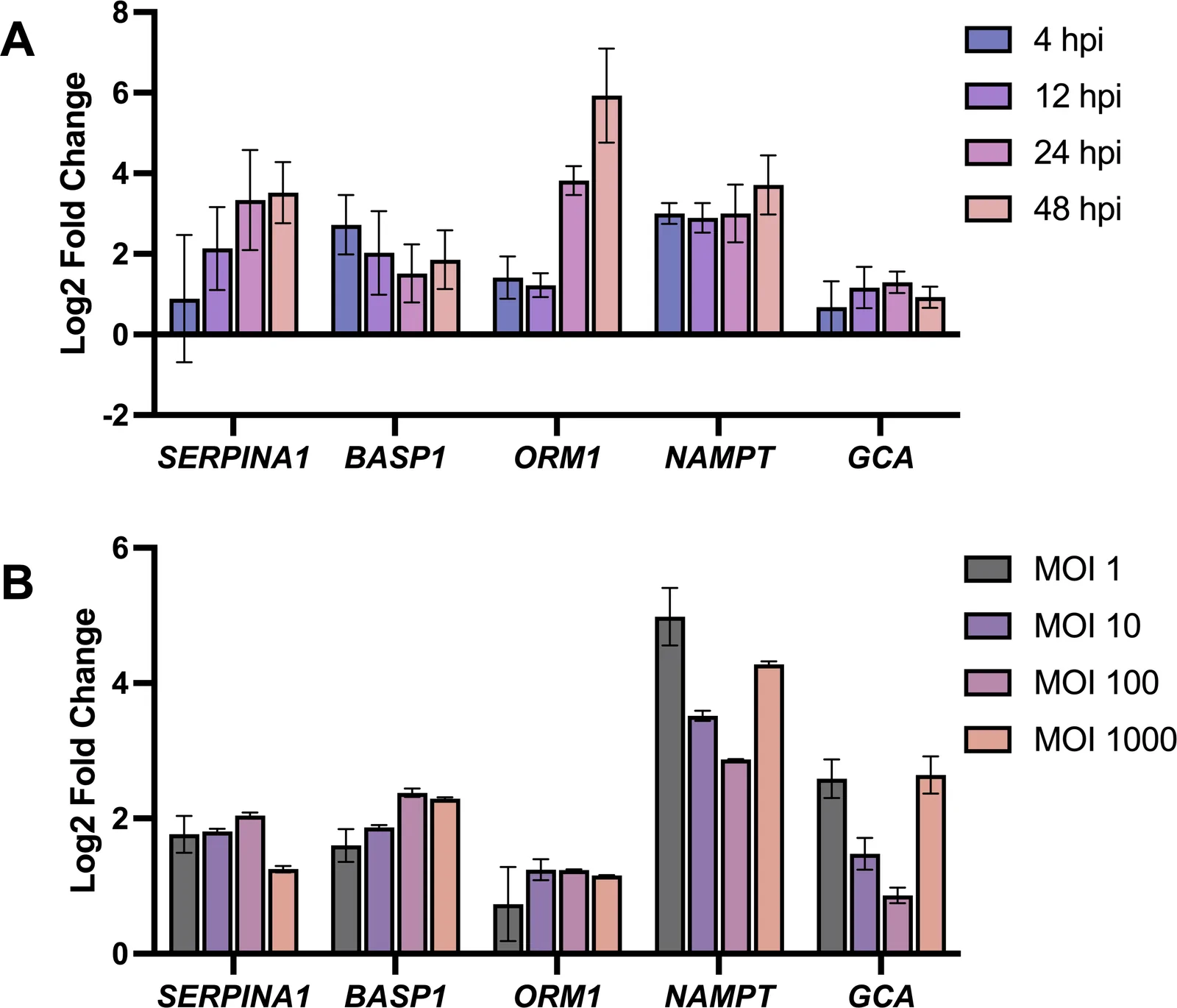
Time- and dose-dependent host gene expression in *Leptospira*-infected THP-1 macrophages. (**A**) Time-course expression of *SERPINA1, BASP1, ORM1, NAMPT,* and *GCA* at 4, 12, 24, and 48 h after infection with live *Leptospira* (MOI 100). (**B**) Dose-response expression of the same genes following 24 h infection at MOIs of 1, 10, 100, and 1,000. Bars represent mean log₂ fold change (ΔΔCt) ±SEM from two biological replicates.

**Fig 8:**
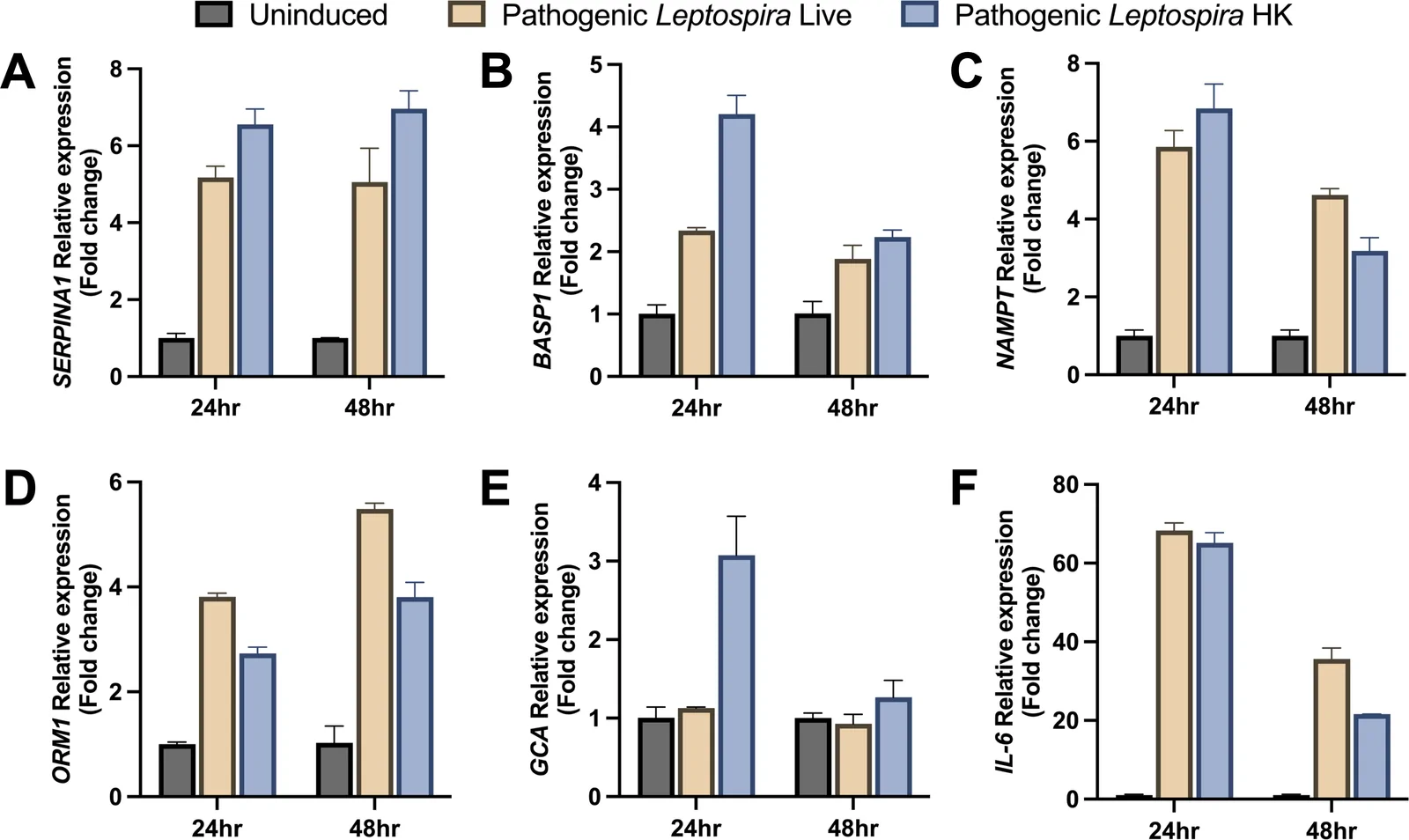
Paracrine induction of host genes via supernatants from *Leptospira*-infected macrophages. Naive THP-1 macrophages were treated with supernatants from uninfected, live *Leptospira*-infected, or heat-killed *Leptospira*-infected THP-1 macrophages. Gene expression of (**A**) *SERPINA1*, (**B**) *BASP1*, (**C**) *NAMPT*, (**D**) *ORM1,* (**E**) *GCA,* and (**F**) *IL-6* was measured by qRT-PCR at 24 h and 48 h. Bars represent mean log₂ fold change ± SD of 2 biological experiments.

Candidate genes for qRT-PCR were selected based on their consistent upregulation in both transcriptomic and proteomic data sets, as well as their relevance to innate immune activation. To ensure robustness across cellular contexts, validation was performed in human whole blood, THP-1 monocytes, and PMA-differentiated THP-1 macrophages with live *L. interrogans* serovar Icterohaemorrhagiae (strain RGA), HK-Lep, or *E. coli* LPS (see Fig. S3 at https://doi.org/10.5281/zenodo.17519428). In human whole blood, live *Leptospira* triggered a robust transcriptional response, with *ORM1, BASP1, SERPING1, OLFM4, FPR2*, and *SERPINA1* being the most highly upregulated genes (Fig. 6A). The response to HK-Lep was attenuated but still showed induction of *SERPINA1, NAMPT, and LBP*, while *E. coli* LPS strongly upregulated *SERPING1, LBP, and NAMPT*. The response was markedly cell-type specific. In THP-1 macrophages, live bacteria induced strong expression of *BASP1, NAMPT, ORM1, SERPINA1,* and *GCA* (Fig. 6B). In contrast, THP-1 monocytes exhibited a more selective response, with pronounced upregulation of *SERPINA1, NAMPT,* and *GCA,* while *BASP1* was notably absent (Fig. 6C). Based on consistent induction and distinct activation patterns, five key genes, namely *NAMPT, BASP1, ORM1, GCA,* and *SERPINA1*, were selected for further analysis. Temporal profiling in THP-1 macrophages revealed differential expression kinetics: *NAMPT, BASP1,* and *GCA* acted as early-response genes, while *ORM1* and *SERPINA1* showed gradual upregulation over 48 h (Fig. 7A). Dose-response analysis indicated that *SERPINA1* and *ORM1* were highly sensitive, reaching maximal induction even at low bacterial loads, whereas *BASP1* expression increased proportionally with the MOI. *NAMPT* and *GCA* exhibited a biphasic pattern, initially decreasing at moderate MOI but upregulated again at high bacterial doses, suggesting complex regulatory control (Fig. 7B). To assess whether these responses were mediated by soluble factors, naive macrophages were exposed to conditioned supernatants from infected cultures. Supernatant from live *Leptospira*-infected macrophages significantly induced *SERPINA1, ORM1,* and *NAMPT* expression in recipient cells. HK-conditioned media also upregulated *SERPINA1* and *BASP1* (Fig. 8). Notably, *GCA* was not induced by conditioned media, confirming that its activation requires direct bacterial contact. Collectively, these results define a core set of dynamically regulated host factors with cell-specific and paracrine-inducible expression profiles during *Leptospira* infection, highlighting their potential roles in shaping early immune and inflammatory responses.

## DISCUSSION

Understanding the host response to *Leptospira* infection is essential to explain the wide spectrum of its clinical manifestations, ranging from mild flu-like symptoms to severe complications such as renal failure and pulmonary hemorrhage. Both genetic and immunological factors influence individual susceptibility to severe disease (1, 29, 30). The immune system controls infection on one hand but also potentially drives immunopathology on the other through exaggerated responses (11, 31–33). Identifying key immune pathways and host biomarkers associated with disease severity can aid in early diagnosis and the development of host-directed therapies (31, 34–36).

Although antibiotic therapy remains the primary treatment for leptospirosis, its effectiveness is largely dependent on early administration and may not fully prevent the severity associated with acute host inflammatory responses. These limitations highlight the importance of understanding host immune response pathways that contribute to disease progression. In this context, our findings provide molecular insights into the host response to *Leptospira* infection, revealing coordinated transcriptional and proteomic alterations in circulating immune cells.

Given the limited availability of clinical samples (*n* = 2 patients, *n* = 2 controls), we interpreted the transcriptomic and proteomic findings in light of the sample size and were further validated using an *ex vivo* whole-blood infection model, *in vitro* infection of human whole blood, THP-1 macrophages, and THP-1 monocytes. Our transcriptomic analysis of PBMCs from leptospirosis patients revealed a clear transcriptional reprogramming, with PCA distinctly separating infected individuals from HCs (PC1 explaining 68.1% variance). Among 5,801 differentially expressed genes, a predominance of upregulated transcripts (4,602) indicated strong immune activation. Enrichment of TNFα signaling via NFκB, interferon α/γ responses, and inflammatory pathways underscores a robust innate immune response, consistent with previous human and experimental studies that highlight cytokine-driven inflammation in leptospirosis (12, 31, 32, 37–39). KEGG and GO analyses further revealed the activation of the complement and coagulation cascades, ECM-receptor interaction, and cytokine-cytokine receptor signaling, suggesting endothelial involvement and vascular remodeling typical of severe disease (40–43). Interestingly, enrichment of *S. aureus* infection pathways may reflect shared antibacterial defense mechanisms (44, 45). In contrast, HCs showed enrichment of oxidative phosphorylation and MYC target genes, indicating metabolic suppression in infected PBMCs suggesting transcriptional alterations in metabolic pathways during infection. Together, our transcriptomic data support the view that leptospiral infection induces widespread inflammatory and metabolic shifts central to disease pathogenesis.

Proteomic profiling of PBMCs from leptospirosis patients revealed substantial alterations in protein expression, with 1,937 proteins identified and 498 DEPs shared across both infected individuals (Fig. 3D). Despite the clear separation of infected and control samples in PCA, the proteomic profiles displayed greater inter-individual variability compared to the transcriptomic data, reflecting potential heterogeneity in disease severity, immune response, or host genetic background (26, 46–48). This variability has been noted in other human infectious disease proteomics studies, including those on dengue and tuberculosis, where individual host responses diverged despite the presence of a common pathogen (26, 47, 49). Furthermore, the proteomic profiling revealed differentially expressed proteins, with enrichment in pathways related to innate immune responses, inflammation, and complement activation (46–48, 50). Several of these proteins, including LCN2, ORM1, SERPINA1, and MMP9, have been previously associated with bacterial infections and sepsis, supporting their relevance in leptospiral pathogenesis and reinforcing their diagnostic and mechanistic significance (31, 47, 51–55). The partial overlap between transcriptomic and proteomic findings also underscores the coordinated regulation at both transcriptional and post-transcriptional levels, reflecting the complex host response to *Leptospira* infection.

To strengthen our findings, we integrated transcriptomic and proteomic data from the same patient samples. This analysis revealed 109 concordantly regulated host factors, of which 52 were consistently upregulated at both RNA and protein levels. This convergence increases confidence in their biological relevance and underscores their potential roles in disease pathogenesis. Reactome pathway analysis of the commonly upregulated factors highlighted key immune pathways, such as neutrophil degranulation, innate immunity, and hemostasis processes, which are central to host defense and inflammation (Fig. 4C) (46, 47, 50, 56). Disease enrichment analysis via DisGeNET linked these molecules to conditions such as sepsis, systemic infection, and vascular inflammation, mirroring the clinical features of leptospirosis (Fig. 4D). Notably, a high-confidence protein-protein interaction network further highlighted 21 tightly connected proteins as potential regulatory hubs in the host immune response. Several of these, including LCN2, SERPINA1, and MMP9, have been previously implicated in leptospiral infection, supporting their mechanistic relevance and utility in further studies (47, 51, 57). Overall, this integrative proteomic and transcriptomic approach provides a comprehensive view of the host response to *Leptospira*, revealing conserved immune pathways and critical regulatory hubs, and establishes a foundation for mechanistic studies and host-directed therapeutic strategies.

To validate the molecular signatures observed in clinical leptospirosis cases, we employed a whole-blood infection model using three *L*. interrogans serovars (Icterohaemorrhagiae, Pomona, and Hardjo). This *ex vivo* system recapitulates the early bloodstream phase of infection and provides a physiologically relevant platform to examine host responses (31, 38, 58). Proteomic profiling of PBMCs post-infection identified 1,036 DEPs, with PCA showing clear separation between infected and control samples. While some serovar-specific differences were noted, the substantial overlap in DEPs indicates a conserved core host response, consistent with prior studies reporting shared innate immune activation and cytokine responses across pathogenic leptospiral strains (29, 31–33, 58). A comparative analysis with clinical PBMC data sets revealed 16 proteins that were consistently upregulated, including APOH, APOB, SERPINA1, and ORM1. Moreover, STRING-based interaction networks highlighted their roles in lipid metabolism, acute-phase responses, and immune modulation, reflecting pathways previously implicated in leptospiral pathogenesis and systemic inflammation (47, 49, 51, 59). Notably, SERPINA1 and ORM1 are established acute-phase reactants that are elevated in severe leptospirosis, suggesting their potential relevance as host-response markers and possible therapeutic targets. The limited overlap among downregulated proteins, with TESC as the only consistently affected factor, may reflect differences in temporal dynamics, post-transcriptional regulation, or cell type-specific responses that are not fully captured in the *ex vivo* system (Fig. 5D) (47, 48, 59, 60). Importantly, the concordance of upregulated proteins between clinical samples and the whole blood model suggests that key innate immune and metabolic pathways are robustly engaged during leptospiral infection, independent of serovar. This aligns with previous findings that leptospiral infection triggers conserved inflammatory signatures, endothelial activation, and acute-phase responses across various hosts (47, 50, 51, 56, 58, 59). Our results thus highlight a 16-protein signature that represents a mechanistic readout of host-*Leptospira* interactions and identifies candidate host-response factors that may support future biomarker discovery.

To validate the host response signature identified through clinical and proteomic analyses, 15 candidate genes were examined by qRT-PCR in an *ex vivo* whole-blood infection model. Seven genes, namely *BASP1, NAMPT, OLFM4, ORM1, SERPINA1, SERPING1,* and *LAMTOR3*, showed consistent upregulation following infection with *L*. interrogans serovars Icterohaemorrhagiae and Pomona. The concordance between transcript and protein profiles highlights their biological relevance in *Leptospira*-induced immune modulation. Several of these genes are known inflammatory mediators. SERPINA1 and ORM1 are acute-phase reactants, whereas OLFM4 and NAMPT indicate neutrophil activation and cellular metabolic stress responses. Their reproducible expression across both clinical and experimental systems suggests that they may be valuable as biomarkers or therapeutic targets.

Functionally, these molecules participate in protease inhibition, hemostasis, and innate immune activation, reflecting a coordinated inflammatory response consistent with earlier reports implicating these pathways in leptospirosis. Among them, SERPINA1 was strongly induced in THP-1 monocytes and macrophages, exhibiting a time- and dose-dependent increase and activation in response to soluble mediators from infected cells. Its product, Alpha-1 Antitrypsin (AAT), is a key serine protease inhibitor that limits tissue injury and modulates the responses of neutrophils and macrophages. The robust induction of SERPINA1 likely represents a host-protective mechanism against excessive inflammation. NAMPT expression was also markedly upregulated in infected macrophages and could be induced by infection-derived supernatants, indicating paracrine signaling. This aligns with the role of extracellular NAMPT (eNAMPT) as a damage-associated molecular pattern (DAMP) that activates TLR4-NFκB signaling and supports NAD^+^ metabolism during infection. Similar to observations in *Mycobacterium tuberculosis* and sepsis, NAMPT likely sustains macrophage energy metabolism and antimicrobial activity during *Leptospira* infection. ORM1 showed a strong, cytokine-driven upregulation, particularly in macrophages. As a classic acute-phase protein, ORM1 modulates inflammation and can promote M2b-like macrophage polarization, potentially aiding in the resolution of infections while also contributing to immune suppression. BASP1 displays cell-specific expression, being transiently upregulated in macrophages but downregulated in monocytes, suggesting early activation of pro-inflammatory signaling that diminishes as infection progresses. GCA was significantly upregulated in both cell types, requiring direct bacterial contact for induction. Known for promoting neutrophil adhesion and amplifying TLR9 signaling, GCA may facilitate early leukocyte recruitment and inflammatory amplification during infection. Together, these findings reveal an integrated host response during *Leptospira* infection, characterized by the upregulation of regulatory proteases (SERPINA1), metabolic enzymes (NAMPT), signaling molecules (BASP1), acute-phase proteins (ORM1), and cell adhesion regulators (GCA). This immune and metabolic pathway signature reflects dynamic innate-adaptive crosstalk and identifies candidate host response factors and pathways that may inform host-directed therapeutic strategies.

In conclusion, our study presents a robust multi-omics framework for identifying key host factors modulated during *Leptospira* infection. By integrating clinical and experimental data, we provide evidence for conserved immune pathways and candidate host response factors associated with *Leptospira* infection that warrant further investigation. However, this study has certain limitations. The clinical sample size was small, and the temporal changes in host responses during different stages of infection were not studied. In addition, the disease specificity and clinical relevance of these candidate host factors were not evaluated and will require further validation in larger patient cohorts, comparative infectious disease data sets, and functional studies using animal models or genetic knockout systems to elucidate their mechanistic roles.

Future research should focus on longitudinal sampling, the inclusion of diverse clinical presentations, and the exploration of host genetic variability to better understand disease severity and outcomes. These efforts may ultimately support the development of improved diagnostic and therapeutic strategies for leptospirosis.

## Data Availability

Raw RNA sequencing data have been deposited in the NCBI Sequence Read Archive under accession number PRJNA1358538. Proteomics data have been deposited in the PRIDE repository under accession number PXD070502. Supplementary figures and tables supporting this study are available via Zenodo at https://doi.org/10.5281/zenodo.17519428. All other data supporting the findings of this study are available from the corresponding author upon reasonable request.

## SUPPLEMENTAL MATERIAL

Supplemental material spectrum.00047-26-s0001.pdf Fig. S1 to S3.

ASM does not own the copyrights to Supplemental Material that may be linked to, or accessed through, an article. The authors have granted ASM a non-exclusive, world-wide license to publish the Supplemental Material files. Please contact the corresponding author directly for reuse.

## References

[1] Haake DA, Levett PN. 2015. Leptospirosis in humans. Curr Top Microbiol Immunol 387:65–97. doi:10.1007/978-3-662-45059-8_5 25388133 PMC4442676

[2] Adler B, de la Peña Moctezuma A. 2010. *Leptospira* and leptospirosis. Vet Microbiol 140:287–296. doi:10.1016/j.vetmic.2009.03.012 19345023

[3] Costa F, Hagan JE, Calcagno J, Kane M, Torgerson P, Martinez-Silveira MS, Stein C, Abela-Ridder B, Ko AI. 2015. Global morbidity and mortality of leptospirosis: a systematic review. PLoS Negl Trop Dis 9:e0003898. doi:10.1371/journal.pntd.0003898 26379143 PMC4574773

[4] Bharti AR, Nally JE, Ricaldi JN, Matthias MA, Diaz MM, Lovett MA, Levett PN, Gilman RH, Willig MR, Gotuzzo E, Vinetz JM, Peru-United States Leptospirosis Consortium. 2003. Leptospirosis: a zoonotic disease of global importance. Lancet Infect Dis 3:757–771. doi:10.1016/s1473-3099(03)00830-2 14652202

[5] Warnasekara J, Srimantha S, Kappagoda C, Jayasundara D, Senevirathna I, Matthias M, Agampodi S, Vinetz JM. 2022. Diagnostic method-based underestimation of leptospirosis in clinical and research settings; an experience from a large prospective study in a high endemic setting. Edited by H. Poonawala. PLoS Negl Trop Dis 16:e0010331. doi:10.1371/journal.pntd.0010331 35377883 PMC9009773

[6] Ko AI, Goarant C, Picardeau M. 2009. *Leptospira*: the dawn of the molecular genetics era for an emerging zoonotic pathogen. Nat Rev Microbiol 7:736–747. doi:10.1038/nrmicro2208 19756012 PMC3384523

[7] Levett PN. 2001. Leptospirosis. Clin Microbiol Rev 14:296–326. doi:10.1128/CMR.14.2.296-326.2001 11292640 PMC88975

[8] Picardeau M. 2017. Virulence of the zoonotic agent of leptospirosis: still terra incognita? Nat Rev Microbiol 15:297–307. doi:10.1038/nrmicro.2017.5 28260786

[9] Nahori M-A, Fournié-Amazouz E, Que-Gewirth NS, Balloy V, Chignard M, Raetz CRH, Saint Girons I, Werts C. 2005. Differential TLR recognition of leptospiral lipid A and lipopolysaccharide in murine and human cells. J Immunol 175:6022–6031. doi:10.4049/jimmunol.175.9.6022 16237097

[10] Werts C. 2018. Interaction of *Leptospira* with the innate immune system. Curr Top Microbiol Immunol 415:163–187. doi:10.1007/82_2017_46 29038956

[11] Reis EAG, Hagan JE, Ribeiro GS, Teixeira-Carvalho A, Martins-Filho OA, Montgomery RR, Shaw AC, Ko AI, Reis MG. 2013. Cytokine response signatures in disease progression and development of severe clinical outcomes for leptospirosis. PLoS Negl Trop Dis 7:e2457. doi:10.1371/journal.pntd.0002457 24069500 PMC3777885

[12] Gomes-Solecki M, Santecchia I, Werts C. 2017. Animal models of leptospirosis: of mice and hamsters. Front Immunol 8:58. doi:10.3389/fimmu.2017.00058 28270811 PMC5318464

[13] Miao H, Chen S, Ding R. 2021. Evaluation of the molecular mechanisms of sepsis using proteomics. Front Immunol 12:733537. doi:10.3389/fimmu.2021.733537 34745104 PMC8566982

[14] Seshadri C, Sedaghat N, Campo M, Peterson G, Wells RD, Olson GS, Sherman DR, Stein CM, Mayanja-Kizza H, Shojaie A, Boom WH, Hawn TR, Tuberculosis Research Unit (TBRU). 2017. Transcriptional networks are associated with resistance to *Mycobacterium tuberculosis* infection. PLoS One 12:e0175844. doi:10.1371/journal.pone.0175844 28414762 PMC5393882

[15] Perera N, Kumar A, Gangadharan B, Ranasinghe D, Wijewickrama A, Malavige GN, Miller JL, Zitzmann N. 2025. Proteomics analysis of peripheral blood mononuclear cells from patients in early dengue infection reveals potential markers of subsequent fluid leakage. Viruses 17:805. doi:10.3390/v17060805 40573396 PMC12197526

[16] Hasin Y, Seldin M, Lusis A. 2017. Multi-omics approaches to disease. Genome Biol 18:83. doi:10.1186/s13059-017-1215-1 28476144 PMC5418815

[17] Babu M, Snyder M. 2023. Multi-omics profiling for health. Mol Cell Proteomics 22:100561. doi:10.1016/j.mcpro.2023.100561 37119971 PMC10220275

[18] Guo M, Xiong M, Peng J, Guan T, Su H, Huang Y, Yang C-G, Li Y, Boraschi D, Pillaiyar T, Wang G, Yi C, Xu Y, Chen C. 2023. Multi-omics for COVID-19: driving development of therapeutics and vaccines. Natl Sci Rev 10:nwad161. doi:10.1093/nsr/nwad161 37936830 PMC10627145

[19] Hadpech S, Thongboonkerd V. 2024. Proteomic investigations of dengue virus infection: key discoveries over the last 10 years. Expert Rev Proteomics 21:281–295. doi:10.1080/14789450.2024.2383580 39049185

[20] Krishnan S, Queiroz ATL, Gupta A, Gupte N, Bisson GP, Kumwenda J, Naidoo K, Mohapi L, Mave V, Mngqibisa R, Lama JR, Hosseinipour MC, Andrade BB, Karakousis PC. 2021. Integrative multi-omics reveals serum markers of tuberculosis in advanced HIV. Front Immunol 12:676980. doi:10.3389/fimmu.2021.676980 34168648 PMC8217878

[21] Chaurasia R, Marroquin AS, Vinetz JM, Matthias MA. 2022. Pathogenic *Leptospira* evolved a unique gene family comprised of ricin b-like lectin domain-containing cytotoxins. Front Microbiol 13:859680. doi:10.3389/fmicb.2022.859680 35422779 PMC9002632

[22] Chen S, Zhou Y, Chen Y, Gu J. 2018. Fastp: an ultra-fast all-in-one FASTQ preprocessor. Bioinformatics 34:i884–i890. doi:10.1093/bioinformatics/bty560 30423086 PMC6129281

[23] Ewels P, Magnusson M, Lundin S, Käller M. 2016. MultiQC: summarize analysis results for multiple tools and samples in a single report. Bioinformatics 32:3047–3048. doi:10.1093/bioinformatics/btw354 27312411 PMC5039924

[24] Dobin A, Davis CA, Schlesinger F, Drenkow J, Zaleski C, Jha S, Batut P, Chaisson M, Gingeras TR. 2013. STAR: ultrafast universal RNA-seq aligner. Bioinformatics 29:15–21. doi:10.1093/bioinformatics/bts635 23104886 PMC3530905

[25] Love MI, Huber W, Anders S. 2014. Moderated estimation of fold change and dispersion for RNA-seq data with DESeq2. Genome Biol 15:550. doi:10.1186/s13059-014-0550-8 25516281 PMC4302049

[26] Kavela S, Vyas P, Cp J, Kushwaha SK, Majumdar SS, Faisal SM. 2023. Use of an integrated multi-omics approach to identify molecular mechanisms and critical factors involved in the pathogenesis of *Leptospira*. Edited by T. Polen. Microbiol Spectr 11:e0313522. doi:10.1128/spectrum.03135-22 36853003 PMC10100824

[27] Perez-Riverol Y, Bandla C, Kundu DJ, Kamatchinathan S, Bai J, Hewapathirana S, John NS, Prakash A, Walzer M, Wang S, Vizcaíno JA. 2025. The PRIDE database at 20 years: 2025 update. Nucleic Acids Res 53:D543–D553. doi:10.1093/nar/gkae1011 39494541 PMC11701690

[28] Tyanova S, Temu T, Sinitcyn P, Carlson A, Hein MY, Geiger T, Mann M, Cox J. 2016. The Perseus computational platform for comprehensive analysis of (prote)omics data. Nat Methods 13:731–740. doi:10.1038/nmeth.3901 27348712

[29] Rajapakse S, Fernando N, Dreyfus A, Smith C, Rodrigo C. 2025. Leptospirosis. Nat Rev Dis Primer 11. doi:10.1038/s41572-025-00614-5 40316520

[30] Esteves SB, Santos CM, Silva BCS, Salgado FF, Guilloux AGA, Cortez A, Lucco RC, Miotto BA. 2023. Time for change? A systematic review with meta-analysis of leptospires infecting dogs to assess vaccine compatibility in Brazil. Prev Vet Med 213:105869. doi:10.1016/j.prevetmed.2023.105869 36773375

[31] Cagliero J, Villanueva SYAM, Matsui M. 2018. Leptospirosis pathophysiology: Into the storm of cytokines. Front Cell Infect Microbiol 8:274770–274770. doi:10.3389/FCIMB.2018.00204/BIBTEX PMC6019470 29974037

[32] Vernel-Pauillac F, Goarant C. 2010. Differential cytokine gene expression according to outcome in a hamster model of leptospirosis. PLoS Negl Trop Dis 4:e582. doi:10.1371/journal.pntd.0000582 20076757 PMC2797601

[33] Fraga TR, Barbosa AS, Isaac L. 2011. Leptospirosis: aspects of innate immunity, immunopathogenesis and immune evasion from the complement system. Scand J Immunol 73:408–419. doi:10.1111/j.1365-3083.2010.02505.x 21204903

[34] Vk C, Ty L, Wf L, Ywy WS, An S, S Z, A M. 2018. Leptospirosis in human: biomarkers in host immune responses. Microbiol Res 207:108–115. doi:10.1016/j.micres.2017.11.015 29458845

[35] Wickramasinghe M, Chandraratne A, Doluweera D, Weerasekera MM, Perera N. 2025. Predictors of severe leptospirosis: a review. Eur J Med Res 30:445. doi:10.1186/s40001-025-02518-2 40457496 PMC12131637

[36] Rajapakse S, Rodrigo C, Handunnetti SM, Fernando SD. 2015. Current immunological and molecular tools for leptospirosis: diagnostics, vaccine design, and biomarkers for predicting severity. Ann Clin Microbiol Antimicrob 14:2. doi:10.1186/s12941-014-0060-2 25591623 PMC4299796

[37] Wang H, Wu Y, Ojcius DM, Yang XF, Zhang C, Ding S, Lin X, Yan J. 2012. Leptospiral hemolysins induce proinflammatory cytokines through toll-like receptor 2-and 4-mediated JNK and NF-κB signaling pathways. PLoS One 7:e42266. doi:10.1371/journal.pone.0042266 22870312 PMC3411626

[38] Senavirathna I, Rathish D, Agampodi S. 2020. Cytokine response in human leptospirosis with different clinical outcomes: a systematic review. BMC Infect Dis 20:268. doi:10.1186/s12879-020-04986-9 32264832 PMC7137275

[39] Faisal SM, Varma VP, Subathra M, Azam S, Sunkara AK, Akif M, Baig MS, Chang Y-F. 2016. Leptospira surface adhesin (Lsa21) induces toll like receptor 2 and 4 mediated inflammatory responses in macrophages. Sci Rep 6:39530. doi:10.1038/srep39530 27996041 PMC5172228

[40] Castiblanco-Valencia MM, Fraga TR, Silva LB da, Monaris D, Abreu PAE, Strobel S, Józsi M, Isaac L, Barbosa AS. 2012. Leptospiral immunoglobulin-like proteins interact with human complement regulators factor H, FHL-1, FHR-1, and C4BP. J Infect Dis 205:995–1004. doi:10.1093/infdis/jir875 22291192

[41] Martinez-Lopez DG, Fahey M, Coburn J. 2010. Responses of human endothelial cells to pathogenic and non-pathogenic *Leptospira* species. PLoS Negl Trop Dis 4:e918. doi:10.1371/journal.pntd.0000918 21179504 PMC3001904

[42] Goeijenbier M, Gasem MH, Meijers JCM, Hartskeerl RA, Ahmed A, Goris MGA, Isbandrio B, Schuller SS, Osterhaus ADME, Martina BEE, van Gorp ECM, Nally JE, Wagenaar JFP. 2015. Markers of endothelial cell activation and immune activation are increased in patients with severe leptospirosis and associated with disease severity. J Infect 71:437–446. doi:10.1016/j.jinf.2015.05.016 26048204

[43] Daroz BB, Fernandes LGV, Cavenague MF, Kochi LT, Passalia FJ, Takahashi MB, Nascimento Filho EG, Teixeira AF, Nascimento ALTO. 2021. A review on host-*Leptospira* interactions: what we know and future expectations. Front Cell Infect Microbiol 11:777709. doi:10.3389/fcimb.2021.777709 34900757 PMC8657130

[44] Kumar S, Lata KS, Sharma P, Bhairappanavar SB, Soni S, Das J. 2019. Inferring pathogen-host interactions between *Leptospira* interrogans and homo sapiens using network theory. Sci Rep 9:1–17. doi:10.1038/s41598-018-38329-1 30723266 PMC6363727

[45] Yan L, Yang Y, Ma X, Wei L, Wan X, Zhang Z, Ding J, Peng J, Liu G, Gou H, Wang C, Zhang X. 2022. Effect of two different drug-resistant *Staphylococcus aureus* strains on the physiological properties of MAC-T cells and their transcriptome analysis. Front Vet Sci 9:818928. doi:10.3389/fvets.2022.818928 35812882 PMC9263607

[46] Novak A, Pupo E, van’t Veld E, Rutten V, Broere F, Sloots A. 2022. Activation of canine, mouse and human TLR2 and TLR4 by inactivated *Leptospira* vaccine strains. Front Immunol 13:1198–1198. doi:10.3389/FIMMU.2022.823058/BIBTEX PMC8978998 35386703

[47] Srivastava R, Ray S, Vaibhav V, Gollapalli K, Jhaveri T, Taur S, Dhali S, Gogtay N, Thatte U, Srikanth R, Srivastava S. 2012. Serum profiling of leptospirosis patients to investigate proteomic alterations. J Proteomics 76 Spec No:56–68. doi:10.1016/j.jprot.2012.04.007 22554907 PMC7185557

[48] Nally JE, Monahan AM, Miller IS, Bonilla-Santiago R, Souda P, Whitelegge JP. 2011. Comparative proteomic analysis of differentially expressed proteins in the urine of reservoir hosts of leptospirosis. PLoS One 6:e26046. doi:10.1371/journal.pone.0026046 22043303 PMC3197145

[49] Fish-Low CY, Than LTL, Ling KH, Lin Q, Sekawi Z. 2020. Plasma proteome profiling reveals differentially expressed lipopolysaccharide-binding protein among leptospirosis patients. J Microbiol Immunol Infect 53:157–162. doi:10.1016/j.jmii.2018.12.015 31029530

[50] Fraga TR, Isaac L, Barbosa AS. 2016. Complement evasion by pathogenic *Leptospira*. Front Immunol 7:623. doi:10.3389/fimmu.2016.00623 28066433 PMC5174078

[51] Vieira ML, Alvarez-Flores MP, Kirchgatter K, Romero EC, Alves IJ, de Morais ZM, Vasconcellos SA, Chudzinski-Tavassi AM, Nascimento ALTO. 2013. Interaction of Leptospira interrogans with human proteolytic systems enhances dissemination through endothelial cells and protease levels. Edited by A. Camilli. Infect Immun 81:1764–1774. doi:10.1128/IAI.00020-13 23478319 PMC3648023

[52] Boix-Palop L, Vergara A, Padilla E, Martínez D, Blanco A, Pérez J, Calbo E, Vila J, Casals-Pascual C. 2023. Evaluation of plasma lipocalin-2 as a predictor of etiology and severity in adult patients with community-acquired pneumonia. Microorganisms 11:1160. doi:10.3390/microorganisms11051160 37317134 PMC10220602

[53] Ruiz M. 2021. Into the labyrinth of the lipocalin α1-acid glycoprotein. Front Physiol 12:686251. doi:10.3389/fphys.2021.686251 34168570 PMC8217824

[54] Nakamura K, Ito I, Kobayashi M, Herndon DN, Suzuki F. 2015. Orosomucoid 1 drives opportunistic infections through the polarization of monocytes to the M2b phenotype. Cytokine 73:8–15. doi:10.1016/j.cyto.2015.01.017 25689617 PMC4541791

[55] Jones TK, Reilly JP, Anderson BJ, Miano TA, Dunn TG, Weisman AR, Agyekum R, Feng R, Ittner CAG, Shashaty MGS, Meyer NJ. 2022. Elevated plasma levels of matrix metalloproteinase-3 and tissue-inhibitor of matrix metalloproteinases-1 associate with organ dysfunction and mortality in sepsis. Shock 57:41–47. doi:10.1097/SHK.0000000000001833 34265829 PMC8663538

[56] Fish-Low CY, Than LTL, Ling KH, Sekawi Z. 2024. The potential of eight plasma proteins as biomarkers in redefining leptospirosis diagnosis. J Proteome Res 23:4027–4042. doi:10.1021/acs.jproteome.4c00376 39150348

[57] Limothai U, Lumlertgul N, Sirivongrangson P, Kulvichit W, Tachaboon S, Dinhuzen J, Chaisuriyong W, Peerapornratana S, Chirathaworn C, Praditpornsilpa K, Eiam-Ong S, Tungsanga K, Srisawat N. 2021. The role of leptospiremia and specific immune response in severe leptospirosis. Sci Rep 11:14630. doi:10.1038/s41598-021-94073-z 34272435 PMC8285422

[58] Goris MGA, Wagenaar JFP, Hartskeerl RA, van Gorp ECM, Schuller S, Monahan AM, Nally JE, van der Poll T, van ’t Veer C. 2011. Potent innate immune response to pathogenic *Leptospira* in human whole blood. PLoS One 6:e18279. doi:10.1371/journal.pone.0018279 21483834 PMC3069077

[59] Novak A, Pennings JLA, van der Maas L, Meiring HD, Ludwig I, Verkoeijen S, Rutten V, Broere F, Sloots A. 2022. Transcriptome and proteome analysis of innate immune responses to inactivated *Leptospira* and bivalent *Leptospira* vaccines in canine 030-D cells. Sci Rep 12:13418. doi:10.1038/s41598-022-16457-z 35927283 PMC9352656

[60] Kolobynina KG, Solovyova VV, Levay K, Rizvanov AA, Slepak VZ. 2016. Emerging roles of the single EF-hand Ca^2+^ sensor tescalcin in the regulation of gene expression, cell growth and differentiation. J Cell Sci 129:3533–3540. doi:10.1242/jcs.191486 27609838 PMC5087652

