# Supplementary material for: Multi-omics analysis of human patient samples identifies key immune factors in Leptospira infection
Source: Microbiol Spectr. 2026 Jun 15;14(7):e00047-26. doi: 10.1128/spectrum.00047-26 (PMC13340129; doi:10.1128/spectrum.00047-26)
Supplement: Supplemental material — Fig. S1 to S3. [file spectrum.00047-26-s0001.pdf]

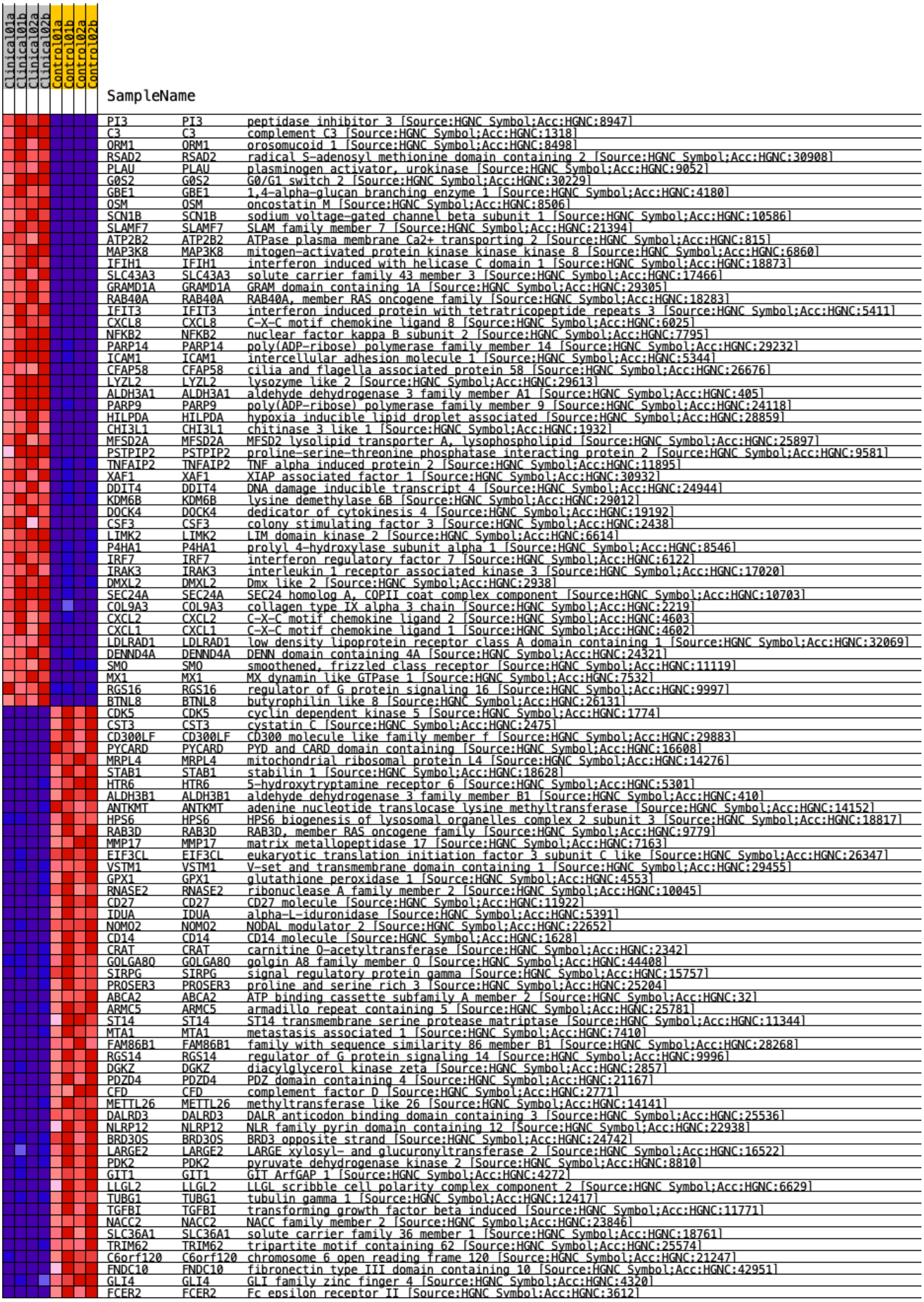

SampleName		
PI3	PI3	peptidase inhibitor 3 [Source:HGNC Symbol;Acc:HGNC:8947]
C3	C3	complement C3 [Source:HGNC Symbol;Acc:HGNC:1318]
ORM1	ORM1	orosomucoid 1 [Source:HGNC Symbol;Acc:HGNC:8498]
RSAD2	RSAD2	radical S-adenosyl methionine domain containing 2 [Source:HGNC Symbol;Acc:HGNC:30908]
PLAU	PLAU	plasminogen activator, urokinase [Source:HGNC Symbol;Acc:HGNC:9052]
G0S2	G0S2	G0/G1 switch 2 [Source:HGNC Symbol;Acc:HGNC:30229]
GBE1	GBE1	1,4-alpha-glucan branching enzyme 1 [Source:HGNC Symbol;Acc:HGNC:4180]
OSM	OSM	oncostatin M [Source:HGNC Symbol;Acc:HGNC:8506]
SCN1B	SCN1B	sodium voltage-gated channel beta subunit 1 [Source:HGNC Symbol;Acc:HGNC:10586]
SLAMF7	SLAMF7	SLAM family member 7 [Source:HGNC Symbol;Acc:HGNC:21394]
ATP2B2	ATP2B2	ATPase plasma membrane Ca2+ transporting 2 [Source:HGNC Symbol;Acc:HGNC:815]
MAP3K8	MAP3K8	mitogen-activated protein kinase kinase kinase 8 [Source:HGNC Symbol;Acc:HGNC:6860]
IFIH1	IFIH1	interferon induced with helicase C domain 1 [Source:HGNC Symbol;Acc:HGNC:18873]
SLC43A3	SLC43A3	solute carrier family 43 member 3 [Source:HGNC Symbol;Acc:HGNC:17466]
GRAMD1A	GRAMD1A	GRAM domain containing 1A [Source:HGNC Symbol;Acc:HGNC:29305]
RAB40A	RAB40A	RAB40A, member RAS oncogene family [Source:HGNC Symbol;Acc:HGNC:18283]
IFIT3	IFIT3	interferon induced protein with tetratricopeptide repeats 3 [Source:HGNC Symbol;Acc:HGNC:5411]
CXCL8	CXCL8	C-X-C motif chemokine ligand 8 [Source:HGNC Symbol;Acc:HGNC:6025]
NFKB2	NFKB2	nuclear factor kappa B subunit 2 [Source:HGNC Symbol;Acc:HGNC:7795]
PARP14	PARP14	poly(ADP-ribose) polymerase family member 14 [Source:HGNC Symbol;Acc:HGNC:29232]
ICAM1	ICAM1	intercellular adhesion molecule 1 [Source:HGNC Symbol;Acc:HGNC:5344]
CFAP58	CFAP58	cilia and flagella associated protein 58 [Source:HGNC Symbol;Acc:HGNC:26676]
LYZL2	LYZL2	lysozyme like 2 [Source:HGNC Symbol;Acc:HGNC:29613]
ALDH3A1	ALDH3A1	aldehyde dehydrogenase 3 family member A1 [Source:HGNC Symbol;Acc:HGNC:405]
PARP9	PARP9	poly(ADP-ribose) polymerase family member 9 [Source:HGNC Symbol;Acc:HGNC:24118]
HILPDA	HILPDA	hypoxia inducible lipid droplet associated [Source:HGNC Symbol;Acc:HGNC:28859]
CHI3L1	CHI3L1	chitinase 3 like 1 [Source:HGNC Symbol;Acc:HGNC:1932]
MFSD2A	MFSD2A	MFSD2 lysolipid transporter A, lysophospholipid [Source:HGNC Symbol;Acc:HGNC:25897]
PSTPIP2	PSTPIP2	proline-serine-threonine phosphatase interacting protein 2 [Source:HGNC Symbol;Acc:HGNC:9581]
TNFAIP2	TNFAIP2	TNF alpha induced protein 2 [Source:HGNC Symbol;Acc:HGNC:11895]
XAF1	XAF1	XIAP associated factor 1 [Source:HGNC Symbol;Acc:HGNC:30932]
DDIT4	DDIT4	DNA damage inducible transcript 4 [Source:HGNC Symbol;Acc:HGNC:24944]
KDM6B	KDM6B	lysine demethylase 6B [Source:HGNC Symbol;Acc:HGNC:29012]
DOCK4	DOCK4	dedicator of cytokinesis 4 [Source:HGNC Symbol;Acc:HGNC:19192]
CSF3	CSF3	colony stimulating factor 3 [Source:HGNC Symbol;Acc:HGNC:2438]
LIMK2	LIMK2	LIM domain kinase 2 [Source:HGNC Symbol;Acc:HGNC:6614]
P4HA1	P4HA1	prolyl 4-hydroxylase subunit alpha 1 [Source:HGNC Symbol;Acc:HGNC:8546]
IRF7	IRF7	interferon regulatory factor 7 [Source:HGNC Symbol;Acc:HGNC:6122]
IRAK3	IRAK3	interleukin 1 receptor associated kinase 3 [Source:HGNC Symbol;Acc:HGNC:17020]
DMXL2	DMXL2	Dmx like 2 [Source:HGNC Symbol;Acc:HGNC:2938]
SEC24A	SEC24A	SEC24 homolog A, COPII coat complex component [Source:HGNC Symbol;Acc:HGNC:10703]
COL9A3	COL9A3	collagen type IX alpha 3 chain [Source:HGNC Symbol;Acc:HGNC:2219]
CXCL2	CXCL2	C-X-C motif chemokine ligand 2 [Source:HGNC Symbol;Acc:HGNC:4603]
CXCL1	CXCL1	C-X-C motif chemokine ligand 1 [Source:HGNC Symbol;Acc:HGNC:4602]
LDLRAD1	LDLRAD1	low density lipoprotein receptor class A domain containing 1 [Source:HGNC Symbol;Acc:HGNC:32069]
DENND4A	DENND4A	DENN domain containing 4A [Source:HGNC Symbol;Acc:HGNC:24321]
SMO	SMO	smoothened, frizzled class receptor [Source:HGNC Symbol;Acc:HGNC:11119]
MX1	MX1	MX dynamin like GTPase 1 [Source:HGNC Symbol;Acc:HGNC:7532]
RGS16	RGS16	regulator of G protein signaling 16 [Source:HGNC Symbol;Acc:HGNC:9997]
BTNL8	BTNL8	butyrophilin like 8 [Source:HGNC Symbol;Acc:HGNC:26131]
CDK5	CDK5	cyclin dependent kinase 5 [Source:HGNC Symbol;Acc:HGNC:1774]
CST3	CST3	cystatin C [Source:HGNC Symbol;Acc:HGNC:2475]
CD300LF	CD300LF	CD300 molecule like family member f [Source:HGNC Symbol;Acc:HGNC:29883]
PYCARD	PYCARD	PYD and CARD domain containing [Source:HGNC Symbol;Acc:HGNC:16608]
MRPL4	MRPL4	mitochondrial ribosomal protein L4 [Source:HGNC Symbol;Acc:HGNC:14276]
STAB1	STAB1	stabilin 1 [Source:HGNC Symbol;Acc:HGNC:18628]
HTR6	HTR6	5-hydroxytryptamine receptor 6 [Source:HGNC Symbol;Acc:HGNC:5301]
ALDH3B1	ALDH3B1	aldehyde dehydrogenase 3 family member B1 [Source:HGNC Symbol;Acc:HGNC:410]
ANTKMT	ANTKMT	adenine nucleotide translocase lysine methyltransferase [Source:HGNC Symbol;Acc:HGNC:14152]
HPS6	HPS6	HPS6 biogenesis of lysosomal organelles complex 2 subunit 3 [Source:HGNC Symbol;Acc:HGNC:18817]
RAB3D	RAB3D	RAB3D, member RAS oncogene family [Source:HGNC Symbol;Acc:HGNC:9779]
MMP17	MMP17	matrix metallopeptidase 17 [Source:HGNC Symbol;Acc:HGNC:7163]
EIF3CL	EIF3CL	eukaryotic translation initiation factor 3 subunit C like [Source:HGNC Symbol;Acc:HGNC:26347]
VSTM1	VSTM1	V-set and transmembrane domain containing 1 [Source:HGNC Symbol;Acc:HGNC:29455]
GPX1	GPX1	glutathione peroxidase 1 [Source:HGNC Symbol;Acc:HGNC:4553]
RNASE2	RNASE2	ribonuclease A family member 2 [Source:HGNC Symbol;Acc:HGNC:10045]
CD27	CD27	CD27 molecule [Source:HGNC Symbol;Acc:HGNC:11922]
IDUA	IDUA	alpha-L-iduronidase [Source:HGNC Symbol;Acc:HGNC:5391]
NOMO2	NOMO2	NODAL modulator 2 [Source:HGNC Symbol;Acc:HGNC:22652]
CD14	CD14	CD14 molecule [Source:HGNC Symbol;Acc:HGNC:1628]
CRAT	CRAT	carnitine O-acetyltransferase [Source:HGNC Symbol;Acc:HGNC:2342]
GOLGA8O	GOLGA8O	golgin A8 family member O [Source:HGNC Symbol;Acc:HGNC:44408]
SIRPG	SIRPG	signal regulatory protein gamma [Source:HGNC Symbol;Acc:HGNC:15757]
PROSER3	PROSER3	proline and serine rich 3 [Source:HGNC Symbol;Acc:HGNC:25204]
ABCA2	ABCA2	ATP binding cassette subfamily A member 2 [Source:HGNC Symbol;Acc:HGNC:32]
ARMC5	ARMC5	armadillo repeat containing 5 [Source:HGNC Symbol;Acc:HGNC:25781]
ST14	ST14	ST14 transmembrane serine protease matriptase [Source:HGNC Symbol;Acc:HGNC:11344]
MTA1	MTA1	metastasis associated 1 [Source:HGNC Symbol;Acc:HGNC:7410]
FAM86B1	FAM86B1	family with sequence similarity 86 member B1 [Source:HGNC Symbol;Acc:HGNC:28268]
RGS14	RGS14	regulator of G protein signaling 14 [Source:HGNC Symbol;Acc:HGNC:9996]
DGKZ	DGKZ	diacylglycerol kinase zeta [Source:HGNC Symbol;Acc:HGNC:2857]
PDZD4	PDZD4	PDZ domain containing 4 [Source:HGNC Symbol;Acc:HGNC:21167]
CFD	CFD	complement factor D [Source:HGNC Symbol;Acc:HGNC:2771]
METTL26	METTL26	methyltransferase like 26 [Source:HGNC Symbol;Acc:HGNC:14141]
DALRD3	DALRD3	DALR anticodon binding domain containing 3 [Source:HGNC Symbol;Acc:HGNC:25536]
NLRP12	NLRP12	NLR family pyrin domain containing 12 [Source:HGNC Symbol;Acc:HGNC:22938]
BRD3OS	BRD3OS	BRD3 opposite strand [Source:HGNC Symbol;Acc:HGNC:24742]
LARGE2	LARGE2	LARGE xylosyl- and glucuronyltransferase 2 [Source:HGNC Symbol;Acc:HGNC:16522]
PDK2	PDK2	pyruvate dehydrogenase kinase 2 [Source:HGNC Symbol;Acc:HGNC:8810]
GIT1	GIT1	GIT ArfGAP 1 [Source:HGNC Symbol;Acc:HGNC:4272]
LLGL2	LLGL2	LLGL scribble cell polarity complex component 2 [Source:HGNC Symbol;Acc:HGNC:6629]
TUBG1	TUBG1	tubulin gamma 1 [Source:HGNC Symbol;Acc:HGNC:12417]
TGFBI	TGFBI	transforming growth factor beta induced [Source:HGNC Symbol;Acc:HGNC:11771]
NACC2	NACC2	NACC family member 2 [Source:HGNC Symbol;Acc:HGNC:23846]
SLC36A1	SLC36A1	solute carrier family 36 member 1 [Source:HGNC Symbol;Acc:HGNC:18761]
TRIM62	TRIM62	tripartite motif containing 62 [Source:HGNC Symbol;Acc:HGNC:25574]
C6orf120	C6orf120	chromosome 6 open reading frame 120 [Source:HGNC Symbol;Acc:HGNC:21247]
FNDC10	FNDC10	fibronectin type III domain containing 10 [Source:HGNC Symbol;Acc:HGNC:42951]
GLI4	GLI4	GLI family zinc finger 4 [Source:HGNC Symbol;Acc:HGNC:4320]
FCER2	FCER2	Fc epsilon receptor II [Source:HGNC Symbol;Acc:HGNC:3612]

Supplementary Figure 1. Heatmap of top 50 differential features from GSEA analysis. Heatmap displaying the 50 most differentially expressed GSEA-identified features across infected (INF) and healthy control (HC) samples. Expression values were collapsed using official gene symbols.

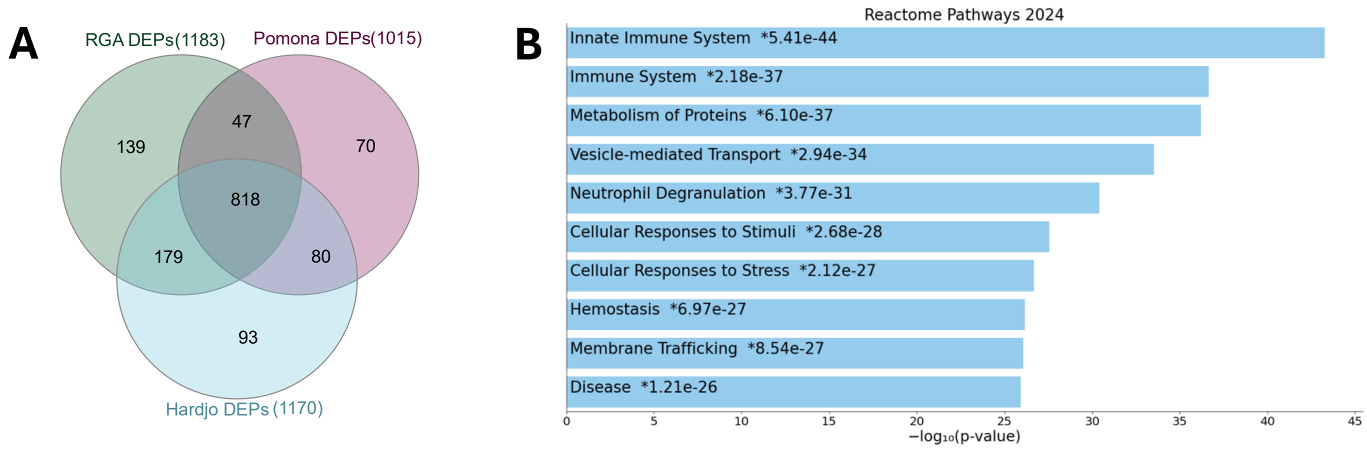

Supplementary Figure 2. Shared proteomic responses among three Leptospira serovars in PBMC infection. (A) Venn diagram showing common DEPs across PBMCs infected with *L. interrogans* serovars RGA, Pomona, and Hardjo. (B) Reactome enrichment analysis of 818 DEPs commonly identified in whole-blood infections with the same serovars.

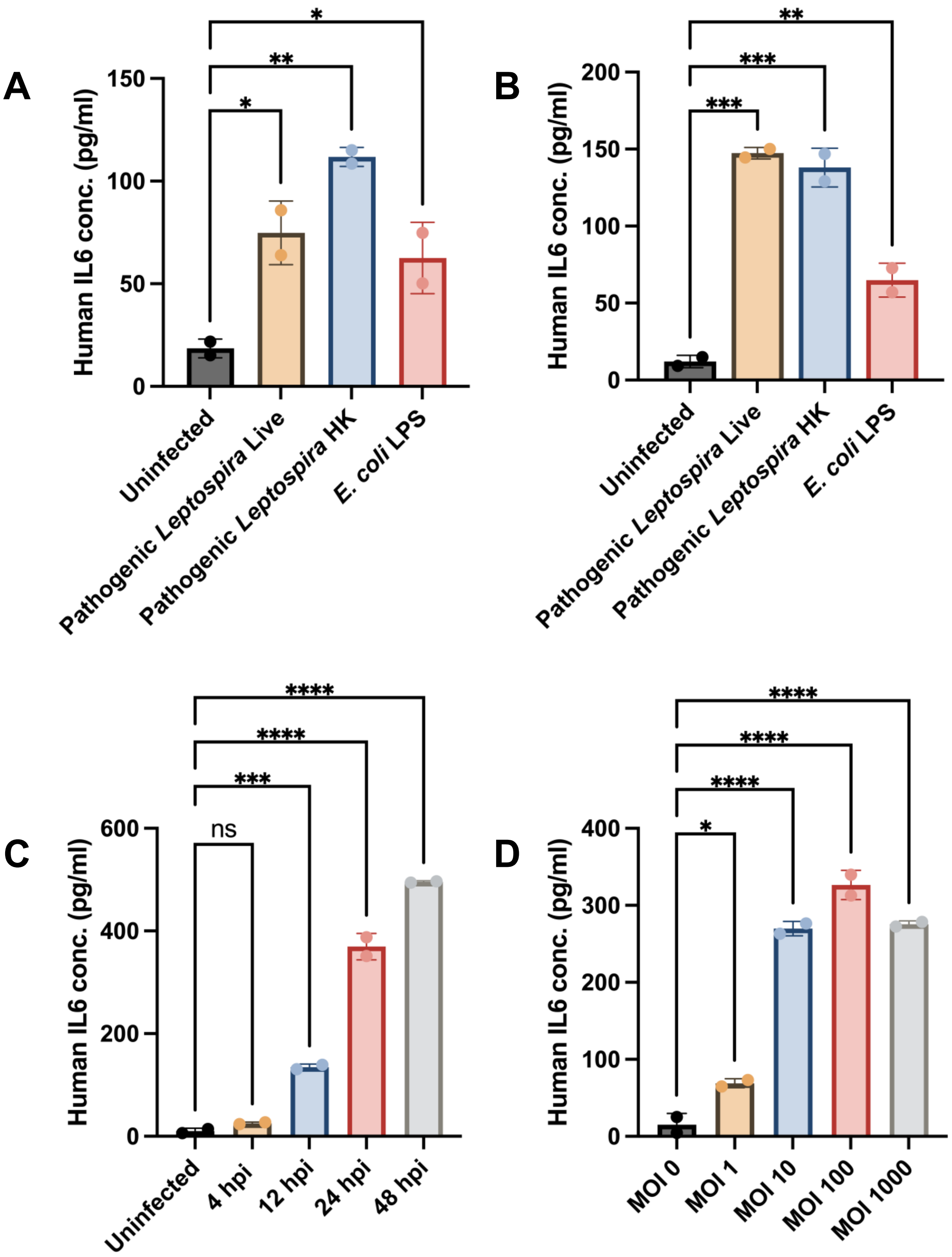

Supplementary Figure 3. IL-6 production in Leptospira-infected THP-1 monocytes and macrophages. IL-6 levels quantified by ELISA from supernatants of (A) THP-1 monocytes (MOI 100, 24 h), (B) THP-1 macrophages (MOI 100, 24 h), (C) macrophages at multiple time points (MOI 100), and (D) macrophages infected at varying MOIs for 24 h. Bars represent mean ± SEM from two independent replicates.
